# The role of the Golden2-like (GLK) transcription factor in regulating terpenoid indole alkaloid biosynthesis in *Catharanthus roseus*

**DOI:** 10.1007/s00299-024-03208-9

**Published:** 2024-05-14

**Authors:** Lauren F. Cole-Osborn, Shannon A. McCallan, Olga Prifti, Rafay Abu, Virginie Sjoelund, Carolyn W. T. Lee-Parsons

**Affiliations:** 1 Department of Chemical Engineering, Northeastern University, Boston, MA 02115 USA; 2 Department of Bioengineering, Northeastern University, Boston, USA; 3 Department of Chemistry and Chemical Biology, Northeastern University, Boston, USA

**Keywords:** GLK, Golden2-like transcription factor, *Catharanthus roseus*, Terpenoid indole alkaloid, Chloroplast retrograde signaling, Lincomycin

## Abstract

**Key message:**

A GLK homologue was identified and functionally characterized in *Catharanthus roseus*. Silencing CrGLK with VIGS or the chloroplast retrograde signaling inducer lincomycin increased terpenoid indole alkaloid biosynthesis.

**Abstract:**

*Catharanthus roseus* is the sole source of the chemotherapeutic terpenoid indole alkaloids (TIAs) vinblastine and vincristine. TIA pathway genes, particularly genes in the vindoline pathway, are expressed at higher levels in immature versus mature leaves, but the molecular mechanisms responsible for this developmental regulation are unknown. We investigated the role of GOLDEN2-LIKE (GLK) transcription factors in contributing to this ontogenetic regulation since GLKs are active in seedlings upon light exposure and in the leaf’s early development, but their activity is repressed as leaves age and senesce.

We identified a GLK homologue in *C. roseus* and functionally characterized its role in regulating TIA biosynthesis, with a focus on the vindoline pathway, by transiently reducing its expression through two separate methods: virus-induced gene silencing (VIGS) and application of chloroplast retrograde signaling inducers, norflurazon and lincomycin. Reducing *CrGLK* levels with each method reduced chlorophyll accumulation and the expression of the light harvesting complex subunit (*LHCB2.2*), confirming its functional homology with GLKs in other plant species. In contrast, reducing *CrGLK* via VIGS or lincomycin increased TIA accumulation and TIA pathway gene expression, suggesting that CrGLK may repress TIA biosynthesis. However, norflurazon had no effect on TIA gene expression, indicating that reducing *CrGLK* alone is not sufficient to induce TIA biosynthesis. Future work is needed to clarify the specific molecular mechanisms leading to increased TIA biosynthesis with *CrGLK* silencing. This is the first identification and characterization of GLK in *C. roseus* and the first investigation of how chloroplast retrograde signaling might regulate TIA biosynthesis.

**Supplementary Information:**

The online version contains supplementary material available at 10.1007/s00299-024-03208-9.

## Introduction

The medicinal plant *Catharanthus roseus* is the exclusive source of the widely used chemotherapeutic medicines, vinblastine and vincristine. Due to the medicinal and economic importance of these terpenoid indole alkaloids (TIAs), researchers have been characterizing the TIA biosynthetic pathway since the 1970s (Mizukami et al. 1979) and signaling pathways that regulate TIA biosynthesis since the 1980s (Balsevich, De Luca, and Kurz, 1986; DeLuca et al. 1986). Through these efforts, *C. roseus* has become a model organism for understanding TIA specialized metabolism.

Monomeric TIAs accumulate to high levels in actively growing immature leaves, but production and accumulation decreases as leaves mature (Besseau et al. 2013; Góngora-Castillo et al. 2012; Mall et al. 2019; Naaranlahti et al. 1991; Qu et al. 2015; B St-Pierre, Vazquez-Flota, & De Luca V, 1999; Benoit St-Pierre, Laflamme, Alarco, D, and Luca, 1998). According to the Optimal Defense Theory, chemical defenses like TIAs accumulate preferentially in high value tissues like immature leaves (Gershenzon and Ullah 2022; Stamp 2003). This ontogenetic pattern is commonly observed (Brown et al. 2003; Gleadow and Woodrow 2000; Ochoa-López et al. 2015; Papazian et al. 2019; Sun et al. 2018; Traw and Feeny 2008), but the underlying molecular mechanisms are just beginning to be explored (Brütting et al. 2017; Meldau et al. 2012). The goal of this paper was to identify a transcription factor involved in the age-related regulation of TIA biosynthesis.

The jasmonate-associated regulation of the upstream TIA pathways leading to strictosidine has been well characterized and involves transcription factors like CrMYC2a, ORCAs, and BISs contributing significantly to their activation (Colinas et al. 2021; Menke et al. 1999; Paul et al. 2017; Schweizer et al. 2018; Singh et al. 2020, 2021; van der Fits and Memelink 2000; Van Moerkercke et al. 2016, 2015). In contrast, the downstream TIA biosynthetic pathways leading to vindoline and catharanthine are not regulated by these factors (Colinas et al. 2021; Schweizer et al. 2018; Singh et al. 2020; Van Moerkercke et al. 2015). Expression of the seven enzymes converting tabersonine to vindoline (T16H2, 16OMT, T3O, T3R, NMT, D4H, DAT: called the vindoline pathway) is not strongly inducible by jasmonate (Aerts et al. 1994; Besseau et al. 2013; Góngora-Castillo et al. 2012; Hernández-Domínguez et al. 2004; D. K. Liscombe et al. 2010; Raina et al. 2012; van der Fits and Memelink 2000; Vázquez-Flota and De Luca 1998; Q. Wang et al. 2010; Wei 2010; Zhou et al. 2015) and is instead activated by light (Yongliang Liu et al. 2019; Schröder et al. 1999; Vazquez-Flota and De Luca 1998; Yu et al. 2018), leaf-specific differentiation (Besseau et al. 2013; Góngora-Castillo et al. 2012; Mall et al. 2019; Qu et al. 2015; Benoit St-Pierre et al. 1998), and developmental state (Besseau et al. 2013; Góngora-Castillo et al. 2012; Mall et al. 2019; Qu et al. 2015; B St-Pierre et al. 1999; Benoit St-Pierre et al. 1998). Transcription factors involved in the regulation of the vindoline pathway are just beginning to be elucidated. Recently, Liu et al. identified CrPIF1 as a transcriptional repressor of the vindoline pathway in the dark and CrGATA1 as an activator of the vindoline pathway in the light (Yongliang Liu et al. 2019).

Working alongside GATAs, Golden2-like (GLK) transcription factors are key activators of chloroplast biogenesis in early seedling and leaf development (Bravo-Garcia, Yasumura, & Langdale, 2009; K. Kobayashi et al. 2012; Koichi Kobayashi et al. 2013; Waters et al. 2008; Waters et al. 2009; Zubo et al. 2018). As etiolated seedlings are transferred to light, GLK is transcribed and translated, activating genes leading to chlorophyll biosynthesis and chloroplast maturation (Bravo-Garcia et al. 2009; Fitter et al. 2002; K. Kobayashi et al. 2012; Koichi Kobayashi et al. 2013; Martin et al. 2016; Song et al. 2014; Waters et al. 2008, 2009; Zhang et al. 2021; Zubo et al. 2018). As leaves age and begin to senesce, GLK transcription and activity is inhibited (Garapati et al. 2015; Rauf et al. 2013; Song et al. 2014). This regulatory pattern of GLK activation in young, light-exposed leaves and GLK inactivation in mature leaves parallels the regulation of the vindoline pathway. Due to these similarities, we hypothesized that GLK may activate the vindoline pathway in *C. roseus*.

The role of GLKs in regulating defense-associated specialized metabolism is limited, but a few studies suggest positive associations between GLKs and disease resistance (X.-Y. Han et al. 2016; Murmu et al. 2014; Savitch et al. 2007). Furthermore, the chloroplast is emerging as a hotspot of defense signaling in addition to its central role in primary metabolism (Kachroo et al. 2021) and GLKs are actively involved in signaling from the chloroplast to the nucleus (i.e. retrograde signaling) (Hills, Khan, & López-Juez, 2015; Kakizaki et al. 2009; Leister & Kleine 2016; Martin et al. 2016; Tokumaru et al. 2017; Waters et al. 2009).

Our study thus explored the potential role of GLK in regulating TIA biosynthesis, with a focus on the vindoline pathway, in *C. roseus*. We identified a GLK homologue in *C. roseus*, CrGLK, and showed that its expression is positively associated with chlorophyll biosynthesis but is negatively associated with TIA biosynthesis. These results contribute to our understanding of TIA pathway regulation and how plants might coordinate chlorophyll and alkaloid biosynthesis during seedling and leaf development.

## Materials and methods

### Identification of CrGLK and CrLHCB2.2

AtGLK1 (NP_565476.1) and AtGLK2 (NP_199232.1) protein sequences were used in a Protein Basic Local Alignment Search Tool (BLASTP) search in the *C. roseus* v.2 translated transcriptome (Franke et al. 2019). The top two hits, CRO_T112335 (41% identity, 99.5% overlap) and CRO_T119410 (72% identity, 18% overlap), were aligned against other GLK, Arabidopsis Response Regulator (ARR), and Arabidopsis Pseudo Response Regulator (APRR) proteins using CLC Main Workbench 21.0.3 (default parameters: gap open cost = 10, gap extension cost = 1). Protein sequences used in the alignment were downloaded from GenBank with accession numbers: AtGLK1 (*Arabidopsis thaliana*, NP_565476.1); AtGLK2 (*Arabidopsis thaliana*, NP_199232.1); ZmG2 (*Zea mays*, AAK50392.1); ZmGLK1 (*Zea mays*, AAK50391.1); OsGLK1 (*Oryza sativa,* BAD62070.1); OsGLK2 (*Oryza sativa,* BAD81484.1); SlGLK1 (*Solanum lycopersicum,* AFF60404.1); SlGLK2 (*Solanum lycopersicum,* AFN69447.1); AtAPRR2 (*Arabidopsis thaliana,* AT4G18020.1); SlAPRR2-like (*Solanum lycopersicum,* AFX68729.1); CaAPRR2-like (*Capsicum annuum,* AGF37241.1); AtARR1 (*Arabidopsis thaliana,* AEE75875.1, outgroup). Domains were annotated on the amino acid alignment according to Fitter et al. 2002 and Makino et al. 2000 (Fitter et al. 2002; Makino et al. 2000).

To identify a homologue of the Light Harvesting Complex Subunit B2.2 (LHCB2.2) in *C. roseus*, we performed a BLASTP search in the *C. roseus* v.2 translated transcriptome (Franke et al. 2019) with the AtLHCB2.2 protein sequence (AAM13371.1). This search returned one highly homologous protein, CRO_T101917 (89% identity, 100% overlap), which was named as CrLHCB2.2.

### Analysis of tissue-specific RNAseq reads

RNAseq reads (SRP005953) from differing tissue types were downloaded from the European Nucleotide Archive (ENA) and imported into KBase for analysis: Flowers (SRR122239, CRA_AA); Cell Suspension Cultures (MJ 0 h) (SRR122250, CRA_AL); Sterile Seedling (SRR122243, CRA_AE); Mature leaf (SRR122251, CRA_AM); Immature leaf (SRR122252, CRA_AN); Stem (SRR122253, CRA_AO); Root (SRR122254, CRA_AP); Hairy root (SRR122257, CRA_AS) (Góngora-Castillo et al. 2012). Adapters (TruSeq3-SE) were clipped and sequences were trimmed using Trimmomatic v0.36 default parameters (sliding window size = 4; sliding window minimum quality = 15) (Bolger et al. 2014). Quality of sequences was confirmed using FastQC v0.11.5 (Wingett & Andrews 2018). Trimmed sequences were aligned to the *C. roseus* genome v. 2 (Franke et al. 2019) using HISAT2 – v2.1.0 with default parameters (Kim et al. 2015). Transcripts were assembled and Transcripts per Million (TPM) values calculated using StringTie with default parameters (Pertea et al. 2015). The KBase narrative can be accessed at 10.25982/95510.53/2310383. Z-scores ((value–mean)/standard deviation) were calculated for the following genes in the varying tissue types: *T16H2*, CRO_T110598; *16OMT*, CRO_T110596; *T3O*, CRO_T113994; *T3R*, CRO_T124298; *NMT*, CRO_T111273; *D4H*, CRO_T127167; *DAT*, CRO_T120021; *CrGLK*, CRO_T112335; *CrGATA1*, CRO_T134526; *G10H*, CRO_T133061; *TDC*, CRO_T125328; *STR*, CRO_T125329; *CS/HL1*, CRO_T139139.

### Gateway cloning for virus-induced gene silencing

For silencing experiments, two fragments from *CrGLK* (Fragment 1 = 188 bp, 519 – 706 from start codon; Fragment 2 = 216 bp, 1040 – 1255 from start codon) were amplified from either *C. roseus* cDNA or a previously cloned plasmid using primers listed in Table S1. Fragments were gel extracted and then cloned into pDONR221 and pTRV2-GATEWAY (Yule Liu et al. 2002) using Gateway^®^ Cloning (Invitrogen). As a positive silencing control, a 499 bp fragment (2450 – 2948 from start codon) of the protoporphyrin IX Magnesium Chelatase Subunit H (*CHLH*) (David K Liscombe & O’Connor 2011) was amplified from *C. roseus* cDNA and cloned into pTRV2-GATEWAY. As a negative non-targeting control, a 471 bp fragment (436 – 906 from start codon) of the Green Fluorescent protein (*GFP*) was amplified from pPD95_77 (Addgene plasmid #1495) and cloned into pTRV2-GATEWAY. This fragment was checked for off-targets by using it as a query sequence in the Sol Genomics Network (SGN) VIGS tool (n-mer = 20, mismatches = 0) (Fernandez-Pozo et al. 2015). No matches in the *C. roseus* v2 transcriptome were found, confirming that the fragment is unlikely to have off-target effects in *C. roseus*. All sequences were confirmed using Sanger Sequencing at Azenta Life Sciences. All plasmids were electroporated into *Agrobacterium tumefaciens* GV3101 (pMP90). pTRV2-CHLH and pTRV2-CrGLK (fragment 1) were deposited at Addgene (IDs: 203,886, 203,888).

### Golden gate modular cloning of CrGLK for overexpression

The *CrGLK* overexpression plasmid and vindoline pathway promoter reporter plasmids were constructed using Golden Gate Modular Cloning. Specific parts are from the MoClo toolkit (Addgene Kit #1,000,000,044) (Weber, Engler, Gruetzner, Werner, & Marillonnet, 2011) or MoClo Plant Parts Kit (Addgene Kit #1,000,000,047) (Engler et al. 2014) unless otherwise noted.

The *CrGLK* coding sequence (CRO_T112335) was amplified from *C. roseus* var. Little Bright Eye cDNA prepared from seedlings using Phusion High-Fidelity DNA Polymerase (New England BioLabs) and primers listed in Table S1. One silent mutation was introduced into the coding sequence of *CrGLK* to remove a BpiI recognition site and domesticate the sequence for Golden Gate cloning (Table S2). Amplified fragments were visualized on agarose gels and then purified using the Zymoclean™ Gel DNA Recovery Kit. Fragments were ligated into the pICH41308 level zero (L0) CDS backbone, and the coding sequence was confirmed with Sanger sequencing at Azenta Life Sciences. The *CrGLK* coding sequence was then amplified without a stop codon from this plasmid and moved into the pAGM1287 level zero (L0) CDS1ns backbone to allow addition of C-terminal tags in the future, if needed.

To facilitate future cloning of coding sequences with and without C-terminal tags, we constructed a pAGM1301 L0 CT plasmid containing a stop codon, as well as a few extra base-pairs that add a glycine and serine to the C-terminus of a CDS to allow fusion to occur. We constructed a transcriptional unit expressing *CrGLK* in the pICH47732 Level 1 Forward position 1 vector backbone consisting of the cauliflower mosaic virus (CaMV) 2 × 35S promoter, the tobacco mosaic virus (TMV) omega 5’UTR, the *CrGLK* CDS1ns, a stop codon (with an additional glycine and serine at the C-terminus), and the *Agrobacterium tumefaciens MAS* terminator.

This transcriptional unit was moved into the pSB90 backbone (Addgene plasmid #123,187), which includes a *VirGN54D* gene in the plasmid backbone to enhance *Agrobacterium* virulence (Mortensen et al. 2019). As a negative control, pSB161 (Addgene plasmid #123,197) was used, which contains *Beta-glucuronidase* (*GUS*) with an intron under control of the same promoter, 5’UTR, and terminator as for *CrGLK* (Mortensen et al. 2019).

After preliminary experiments suggesting that CrGLK was inactive when overexpressed, out of precaution, glycine and serine at the C-terminus of CrGLK, artifacts of cloning the stop codon separately, were removed from the final L2 plasmid using the Q5® Site-Directed Mutagenesis Kit (NEB). The transcriptional unit in this plasmid was again sequence-confirmed with Sanger sequencing.

L2 plasmids were electroporated into *Agrobacterium tumefaciens* GV3101 (pMP90). L0 was deposited at Addgene (IDs: 203,903 – 203,906).

### Golden gate modular cloning of vindoline pathway promoters for sequence confirmation

The promoters of the vindoline pathway genes with their 5’UTRs (approximately 1 kb upstream of start codon) were amplified in parts using Phusion High-Fidelity DNA Polymerase (New England BioLabs) and sequenced from *C. roseus* var. Little Bright Eye gDNA using primers listed in Table S1. Mutations were introduced to mutate BpiI and BsaI recognition sites and domesticate the sequences for Golden Gate Modular Cloning (Table S2). Some of these sequences (pT16H2, p16OMT, pT3O, and pD4H) differed from the sequences predicted from the *C. roseus* genome v.2. The genome was sequenced from the Sunstorm Apricot cultivar, so the discrepancies likely arose from cultivar differences. Amplified promoter + 5’UTR sequences were deposited in Genbank (Accessions: OR052132-OR052138). Sequences were cloned into the pICH41295 L0 promoter + 5’UTR vector and deposited at Addgene (IDs: 203,889 – 203,895).

### Virus-induced gene silencing (VIGS)

*C. roseus* var. Little Bright Eye seeds (NESeeds, 0.4 g) were sterilized by submersion in 70% ethanol for 45 s, 30% bleach and 1X Triton for 6 min, triple-rinsed in sterile water, and incubated in 3% Plant Preservative Mixture (PPM) for 18 h in the dark. The PPM was decanted, and the seeds were spread on full-strength Gamborg’s media (3.1 g/L Gamborg’s basal salts, 1 ml/L Gamborg’s 1000X vitamins, and 6% micropropagation agar type 1, Phytotechnology Laboratory) inside a sterile Magenta^™^ Plant Culture Box (Sigma) for germination. Seeds were germinated in the dark at 25–27 °C until seedlings were about 2 cm tall (about 7 days). Seedlings were then transferred to 16-h light/8-h dark photoperiods (red and blue LED lights, ~ 80 µmol m^−2^ s^−1^) for at least two days. Once seedlings had undergone photomorphogenesis, they were planted in soil (Miracle-Gro) in 2.25″ square cells and grown under 16-h light/8-h dark photoperiod (red and blue LED lights, ~ 90 µmol m^−2^ s^−1^) until two true leaves appeared (about 4–6 weeks).

Once seedlings had two true leaves, they were infected with *Agrobacterium tumefaciens* according to the pinch-wounding method (David K Liscombe & O’Connor 2011). A single colony of *A. tumefaciens* GV3101 (pMP90) harboring pTRV1 or pTRV2 was used to inoculate a 10 mL culture of LB with gentamycin (10 mg/L, selects for pMP90) and kanamycin (50 mg/L, selects for pTRV1 and pTRV2-GATEWAY) in a 50 mL conical centrifuge tube. This culture was grown at 26˚C and 250 RPM for two days. It was then pelleted, resuspended in 10 mL of induction media (10.46 g/L *Agrobacterium* minimal medium (PlantMedia), 100 µM acetosyringone) with antibiotics, and grown for another 3 h. It was then pelleted again and resuspended in 1 mL of VIGS infiltration media (10 mM MgSO_4_, 10 mM MES pH 5.8, 200 μM acetosyringone). *A. tumefaciens* strains containing pTRV1 and pTRV2 plasmids were combined in a 1:1 ratio (OD_600_ of each strain = 2–4). Modified tweezers were dipped into the *A. tumefaciens* solution and the plant was pinched three times in the highest internode beneath the shoot apical meristem (dipping into the solution between each pinch).

After infection, plants were kept in the dark for two days before being placed back into a 16-h light/8-h dark photoperiod, either under red and blue LED lights (~ 90 µmol m^−2^ s^−1^) or white, fluorescent lights (~ 15 µmol m^−2^ s^−1^). Light measurements are an average of five measurements taken with the Apogee SQ-520 Full Spectrum Smart Quantum Sensor. Plants were grown until two pairs of leaves emerged after silencing and the CHLH-silenced plant exhibited yellow leaves (about 2–3 weeks). At this point, a single leaf from the two youngest leaf pairs was individually harvested (unless otherwise noted) for RNA extraction.

### Chlorophyll quantification

Using a 1-hole punch, a 6 mm diameter leaf disc (~ 10 mg) was collected from the 1st and 2nd leaf pairs after VIGS infection. Each leaf disc was placed in a 2 mL screwcap microcentrifuge tube containing ten 3 mm glass beads and was flash frozen in liquid nitrogen. Frozen leaf discs were pulverized in a Mini-BeadBeater-16 (Biospec) for 20 s and placed back in liquid nitrogen to keep cold. After 1 mL of 80% acetone was added to the crushed tissue, samples were vortexed briefly, incubated on ice in the dark for 15 min, and then centrifuged at 19,000 RPM for 2 min. Supernatant was transferred to a new 2 mL tube and the extraction was repeated. Supernatants from each extraction were combined. Undiluted supernatant was transferred to a clear-bottom 96-well plate (Greiner Bio-One 65,509) to measure absorbance at 645 nm and 663 nm with a Biotek Synergy HT microplate reader. Chlorophyll content was calculated using the formulas (Ördög & Zoltán, 1949): [Chl-a] = 12.7(A_663_) – 2.69(A_645_) and [Chl-b] = 22.9(A_645_) – 4.68(A_663_) where [Chl-a] and [Chl-b] are in mg/L. Values were converted to µg per cm^2^ using the extraction volume (2 mL) and leaf disc area (0.28 cm^2^). Linear range and extraction efficiency of the assay were validated before analysis.

### Alkaloid extraction

At the time of harvest, individual leaves were weighed to determine their fresh weight (range: 2–180 mg), placed in a 2 mL screw cap tube containing ten 3 mm glass beads and flash-frozen in liquid nitrogen. While still frozen, tissue was crushed by shaking in a Mini-BeadBeater-16 (Biospec) for 15 s. Crushing was repeated four times, placing the samples back on liquid nitrogen between each cycle. Alkaloids were extracted by adding 1 mL of methanol, vortexing, and sonicating samples in an ice bath for 30 min (vortexing every 10 min). Samples were then centrifuged at 10,000 rcf for 10 min. Methanol was removed and the extraction with 1 mL methanol was repeated two more times. Methanol extract was combined for each sample into a 15 mL Falcon tube and evaporated using a SpeedVac Concentrator (Savant SC210A, RVT4104, VN100, VLP200). Immediately prior to analysis, samples were resuspended in 20 mL methanol per gram of fresh weight. Samples were vortexed and sonicated to ensure full resuspension. To ensure that there were no particulates in the samples, they were placed at 4˚C for at least 4 h before being centrifuged at 10,000 rcf for 10 min. Supernatant was transferred to a new tube, and centrifugation was repeated. Finally, extract was diluted 1:50 in 50% methanol/50% water.

### Alkaloid quantification with HPLC–MS/MS

Quantification of the alkaloids was performed at the Mass Spectrometry Facility at Northeastern University. The Thermo Scientific™ Vanquish HPLC system with a Phenomenex Luna Omega LC column (1.6 μm C18 100 A°, 2.1 × 50 mm) was used for alkaloid separation. The mobile phase consisted of 0.1% formic acid in water (solvent A) and 0.1% in acetonitrile (solvent B). The protocol consisted of 15% solvent B for 0.5 min, a gradient of 15–31% solvent B for 15.5 min to elute the alkaloids, and finally 98% solvent B for 3 min. The column was re-equilibrated with 15% solvent B for 4 min prior to the next injection. The flow rate was 0.3 mL/min, the column temperature was maintained at 35 °C, and the injection volume was 1 μL.

The compounds were detected on a Tandem HRMS Orbitrap mass analyzer (Thermo Scientific Exploris 240) coupled to an electrospray ionization (H-ESI) source in the positive mode with typical settings (supplementary materials). Quantitative analysis was performed in the full scan MS with data-dependent tandem mass spectrometry (full MS/dd MS2 mode with inclusion list, supplementary materials). The parent ion and the confirmatory fragment ion at the optimal collision energies (CE) of each alkaloid were catharanthine 337.19 → 144.08 (CE 31.5), ajmalicine 353.19 → 144.08 (CE 30), serpentine 349.16 → 263.08 (CE 46), and vindoline 457.23 → 188.11 (CE 34). The data processing and area under the curve of the extracted ion chromatogram (XIC) was performed on Thermo Scientific Xcalibur Version 4.5.474.0.

Extraction and quantification of alkaloids were validated as follows: three extractions were sufficient to extract more than 95% of total alkaloids from 50 mg dry weight leaf tissue (~ 500 mg fresh weight) with the percent recovery measured at 100 ± 10%. The linear range of the quantified alkaloids was validated with a calibration curve of standards prepared in solvent.

### RNA extraction and quantitative PCR

Expression levels of *CrGLK* and vindoline pathway genes were monitored using quantitative real-time PCR (qRT-PCR) with primers listed in Table S3. mRNA was extracted from liquid nitrogen flash-frozen leaf tissue or seedlings (5 whole seedlings pooled as one biological replicate) placed in a 2 mL screw cap tube containing ten 3 mm glass beads and stored at -80˚C until needed. While still frozen, tissue was crushed by shaking in a Mini-BeadBeater-16 (Biospec) for 15 s. Crushing was repeated twice, placing the samples back on liquid nitrogen between each cycle. Afterwards, RNA was extracted with RNAzol-RT (Molecular Research Center) and the Direct-zol RNA Miniprep Plus Kit (Zymo Research) with on-column DNAse treatment to remove genomic DNA. RNA integrity was assessed using agarose gel electrophoresis. Concentration and purity were quantified with a NanoDrop (ND-1000 Spectrophotometer; ThermoScientific). cDNA was synthesized using either the SuperScript II First-Strand Synthesis System (Invitrogen) or the LunaScript RT SuperMix Kit (New England Biolabs) with up to 2.5 µg of RNA, according to manufacturer’s instructions. cDNA was diluted 1:4, and 1 µL was used in a 10 µL reaction with SYBR Green ROX qPCR Master Mix (Qiagen or ABClonal) and 300 nM primers on the MX3000P (Agilent) or CFX96 (Bio-Rad) qPCR instrument using the following thermocycler protocol: 10 min at 95 °C (Taq activation), 30 s at 95 °C (denaturing), 45 s at 60 °C (annealing), 30 s at 72 °C (extension), steps 2–4 repeated for 40 cycles, followed by a melt curve. For *G10H*, *TDC*, and *STR* primers, concentrations were used according to previous optimizations (G10h-forward = 100 nM; G10h-reverse = 600 nM; Tdc forward = 300 nM; Tdc reverse = 600 nM; Str_F = 100 nM; Str_R = 300 nM) (Goklany et al. 2009; Rizvi et al. 2016). Ct values for each biological replicate were calculated as the average of two technical replicates. Transcript levels were normalized to the housekeeping gene, *SAND* (Pollier et al. 2014), and fold changes relative to the negative control condition were calculated according to the 2^−∆∆Ct^ method (Livak & Schmittgen 2001). Amplification efficiency for each primer set was confirmed using Ct values over a range of cDNA dilutions and was 100 ± 10% for each gene monitored. Specificity of the primers was confirmed by gel electrophoresis and sequencing. *SAND* Ct values in no reverse-transcriptase controls were confirmed to be at least 5 Ct values above the respective experimental sample, indicating minimal genomic DNA contamination (Svec et al. 2015).

### Efficient agro-mediated seedling infiltration (EASI) for transient overexpression of CrGLK

To overexpress *CrGLK*, wild-type *C. roseus* seedlings were transformed according to the efficient *Agro*-mediated seedling infiltration (EASI) method (Mortensen et al. 2019) with an *A. tumefaciens* strain containing a *CaMV2* × *35S* driven *CrGLK* or a *CaMV2* × *35S* driven *GUS* (negative control) at an OD_600_ of 0.2. After infiltration, seedlings were kept in the dark for 2 days and then moved to continuous red and blue LED lights for 24 h prior to harvest. Five seedlings were pooled for each biological replicate.

### Chloroplast retrograde signaling treatments

*C. roseus* var. Little Bright Eye (NESeeds, 0.8 g) was sterilized by incubation in 4% Plant Preservative Mixture (PPM) for 18 h in the dark; the PPM was decanted, and the seeds were spread on full-strength Gamborg’s media with appropriate treatments in Magenta™ Plant Culture Boxes. Lincomycin hydrochloride (Thermo Scientific) was dissolved in water at a concentration of 0.5 M, filter-sterilized, and added to media after autoclaving at a final concentration of 0.5 mM. Norflurazon was dissolved in methanol (Crescent Chemical Co Inc.) at a concentration of 1000 µg/mL and added to media after autoclaving at a final concentration of 5 µM. An equal volume of methanol was added to media for the mock treatment. Norflurazon and mock-treated media were left open in a laminar flow hood for about 1 h to allow residual methanol to evaporate.

After germination in the dark at 25–27 °C for about 9 days, seedlings were transferred to 16-h light/8-h dark photoperiods (red and blue LED lights, Benchmark MyTemp 65HC Digital Cooling Incubator) at 25 °C for two full days. Seedlings were harvested at the beginning of the third day. Five biological replicates were harvested, each containing a pool of five seedlings.

### Statistical analysis

The effect of *CrGLK*-silencing, light intensity, and developmental state (leaf age) on chlorophyll levels, alkaloid levels, and gene expression was analyzed using a univariate general linear model fitted to each dependent variable with IBM SPSS Statistics v. 28.0.0.0. Leaf age was treated as a within-subject repeated measure since two leaves at different developmental states for each plant were measured and a positive correlation can be expected as the two leaves were taken from the same plant. The other factors (*CrGLK-*silencing and light intensity) were treated as independent between-subject fixed factors, as each replicate consisted of an individual plant. For each dependent variable (chlorophyll levels, alkaloid levels, and gene expression), a full factorial linear model was fitted. Type III sum of squares *F*-tests was performed to determine significant contributions to the models.

Fold change in gene expression of lincomycin-treated, norflurazon-treated, mock-treated, or untreated seedlings (Fig. 6, Fig. S4) was compared using a one-way ANOVA performed in JMP Pro 15. The resulting p-values were adjusted for FDR among the 13 genes measured using a two-stage linear step-up procedure of Benjamini, Krieger, and Yekutieli (*Q* = 5%) in GraphPad Prism v. 9.5.1. The Dunnett’s test was performed on genes with significant ANOVAs in JMP Pro 15 with mock-treated as the control condition (Fig. 6) or untreated as the control condition (Fig. S4).

## Results

### C. roseus has a single GLK homologue

GLK proteins are members of the GARP (GOLDEN2, ARR, and Psr1) superfamily of transcription factors that share a conserved DNA binding domain. Other members of this family include the cytokinin-responsive Arabidopsis Response Regulators (ARRs) and Arabidopsis Pseudo Response Regulators (APRRs). GLKs have a unique “AREAEAA” motif that distinguishes them from other GARP transcription factors (Fitter et al. 2002).

**Fig. 1:**
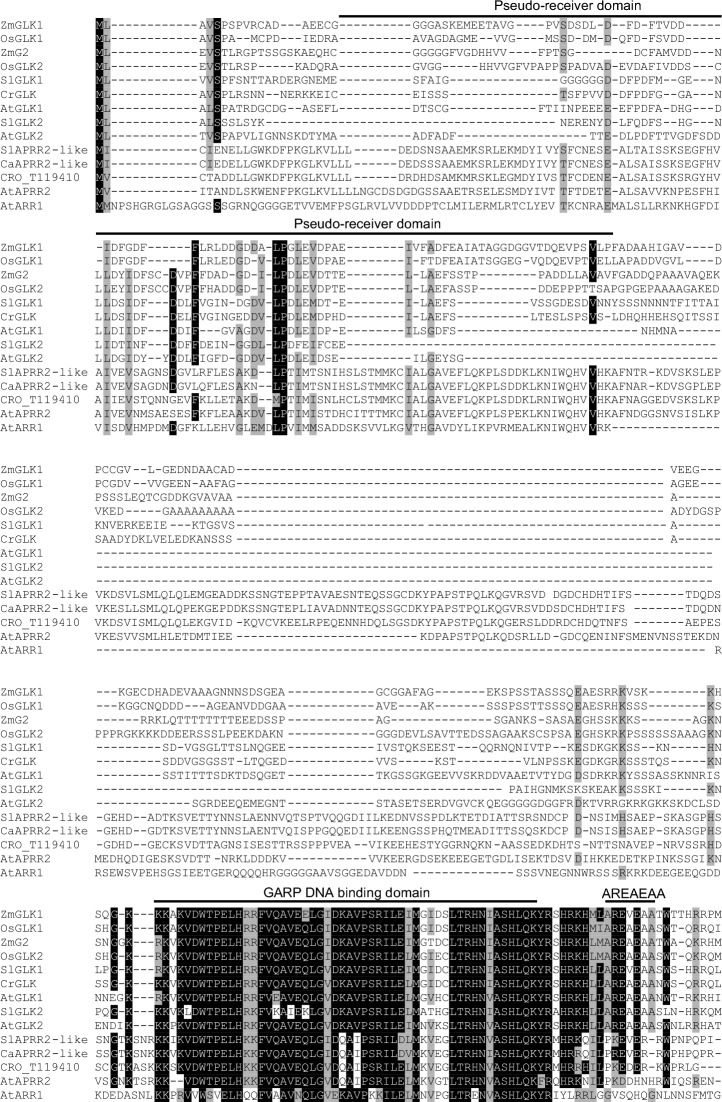

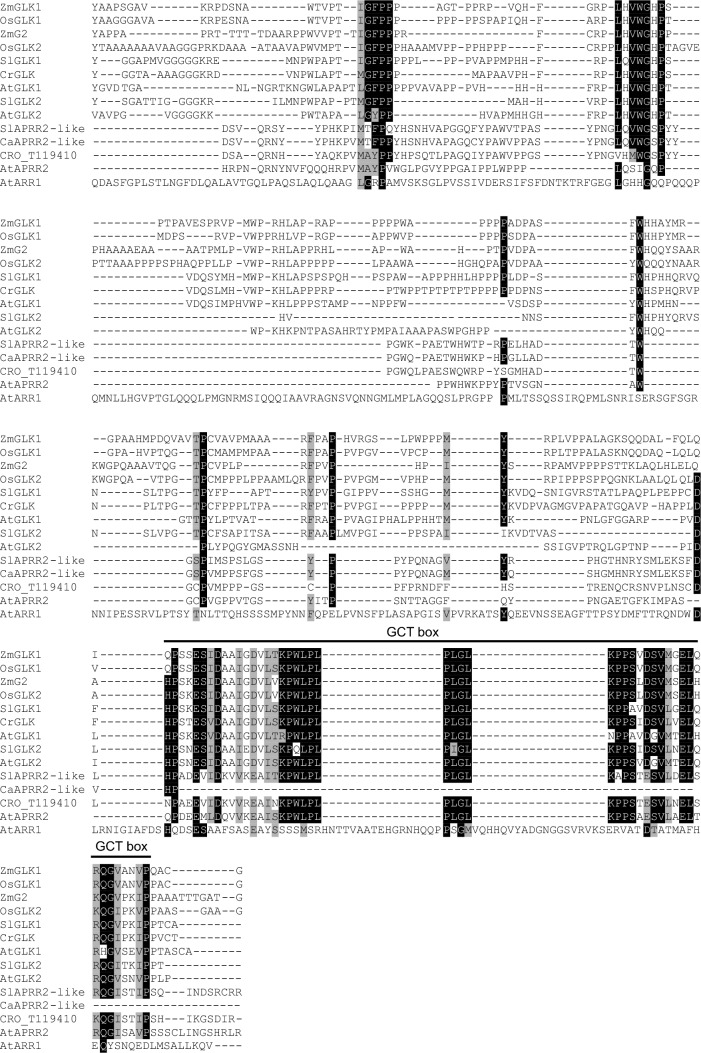
Amino acid alignment of GLK and APRR2-like proteins. Protein sequences were downloaded from GenBank with accession numbers: AtGLK1 (*Arabidopsis thaliana*, NP_565476.1); AtGLK2 (*Arabidopsis thaliana*, NP_199232.1); ZmG2 (*Zea mays*, AAK50392.1); ZmGLK1 (*Zea mays*, AAK50391.1); OsGLK1 (*Oryza sativa*, BAD62070.1); OsGLK2 (*Oryza sativa*, BAD81484.1); SlGLK1 (*Solanum lycopersicum*, AFF60404.1); SlGLK2 (*Solanum lycopersicum*, AFN69447.1); AtAPRR2 (*Arabidopsis thaliana*, AT4G18020.1); SlAPRR2-like (*Solanum lycopersicum*, AFX68729.1); CaAPRR2-like (*Capsicum annuum*, AGF37241.1); AtARR1 (*Arabidopsis thaliana*, AEE75875.1, outgroup). Sequences were aligned with CrGLK (CRO_T112335) and the next closest homologue in *C. roseus* (CRO_T119410) using CLC Main Workbench 21.0.3 (default parameters: gap open cost = 10, gap extension cost = 1). Domains were annotated according to Fitter et al. 2002 and Makino et al. 2000 (Fitter et al. 2002; Makino et al. 2000)

To identify GLK homologues in *C. roseus*, we performed a BLASTP search in the *C. roseus* v.2 translated transcriptome (Franke et al. 2019) using AtGLK1 (NP_565476.1) and AtGLK2 (NP_199232.1) as queries. The top hit for both AtGLK1 and AtGLK2 was CRO_T112335. An amino acid alignment with other GLK proteins showed that this sequence contained the expected GARP DNA binding domain (Fitter et al. 2002; Makino et al. 2000), a GCT box involved in protein–protein interactions (Alem et al. 2022; Y. Han et al. 2024; Rauf et al. 2013; Tachibana et al. 2024; Zhang et al. 2021), and the “AREAEAA” motif of unknown function (Fitter et al. 2002; Makino et al. 2000) (Fig. 1). The second top hit in the BLAST query, CRO_T119410, contained the GARP DNA-binding domain and GCT box but did not contain the highly conserved “AREAEAA” motif. Instead, CRO_T119410 contains an N-terminal pseudo-receiver domain and is most similar to APRR2-like proteins, close relatives to GLK proteins, but defined as a separate family (Fig. 1) (Fitter et al. 2002). This search suggests that there is only one GLK protein (CRO_T112335) expressed in *C. roseus*, which we refer to as CrGLK. Although many plants have two GLK proteins, previous evolutionary analyses suggested that the gene pairs arose through separate duplication events in monocots and dicots, and that all C_4_ plants have duplicate GLKs, but some C_3_ plants have only one GLK (Fitter et al. 2002; P. Wang et al. 2013). *C. roseus* is a C_3_ plant and so our identification of a single GLK is consistent with this evolutionary history.

The *CrGLK* coding sequence was successfully amplified from *C. roseus* seedling cDNA and its sequence was confirmed to match the predicted CRO_T112335 transcript, with a single silent mutation (A-to-C, 1065 bp downstream of start codon).

### Vindoline pathway promoters contain multiple GLK binding motifs

As a first step towards determining whether CrGLK regulates the vindoline pathway, we searched for GLK binding motifs (RGATTYY) (Tu et al. 2022) in vindoline pathway promoters and 5’UTRs. The promoter sequences were extracted from the sequenced genome (Sunstorm Apricot cultivar) and PCR-amplified and sequence-confirmed (Little Bright Eye cultivar). There were some differences in the promoter sequences present in these two cultivars, but there were multiple GLK-binding motifs present in the promoters of both cultivars, suggesting conserved regulatory motifs (Table S4). The presence of multiple GLK binding motifs in the vindoline pathway promoters supported our hypothesis that CrGLK could potentially bind and modulate their expression.

### The expression of CrGLK and vindoline pathway genes were similar with tissue type but dissimilar with light intensity and developmental state

**Fig. 2:**
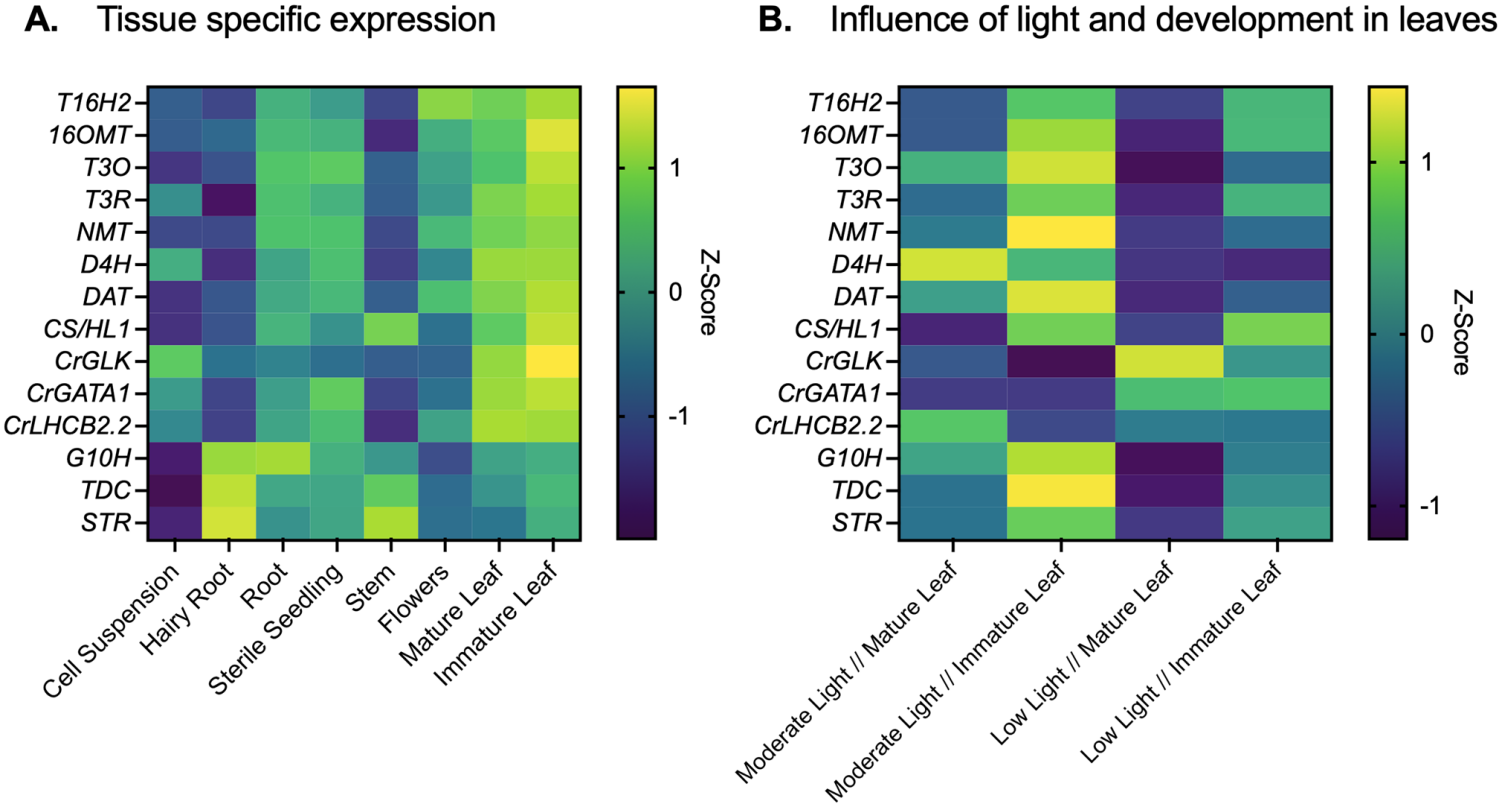
*CrGLK* and TIA gene expression. **A** The tissue specific expression of *CrGLK* is similar to vindoline pathway genes and *CrGATA1*. Z-scores were calculated from TPM values from previously published RNAseq data (Góngora-Castillo et al. 2012). **B** Basal expression levels of *CrGLK, CrGATA1, CrLHCB2.2*, and TIA pathway genes in leaves of varying light intensities and developmental states. Expression levels were calculated from qPCR results of *GFP*-silenced plants (negative VIGS control) calculated using the 2^-∆∆Ct^ method (Livak & Schmittgen 2001) relative to the housekeeping gene, *SAND* (Pollier et al. 2014). RNA was extracted from one mature leaf and one immature leaf for each plant (*N* = 4–5 individual plants). Z-scores were calculated from these relative expression levels for each of the monitored genes

We explored the correlation between the expression of *CrGLK* and the vindoline pathway genes with tissue type, light intensity, and developmental state to probe the role of CrGLK in regulating the vindoline pathway. First, we used previously published RNAseq data (Góngora-Castillo et al. 2012) to investigate the tissue specificity of *CrGLK*. *CrGLK* was most highly expressed in leaves, similar to *CrGATA1,* vindoline pathway genes, and *CS/HL1* (which leads to catharanthine biosynthesis). In contrast, upstream TIA genes (*G10H, TDC, and STR*) were highly expressed in other tissue types like hairy roots and stems. This positive correlation in the tissue specificity of *CrGLK* and the vindoline pathway supported our hypothesis that CrGLK may positively regulate the vindoline pathway in leaves (Fig. 2A).

**Fig. 3:**
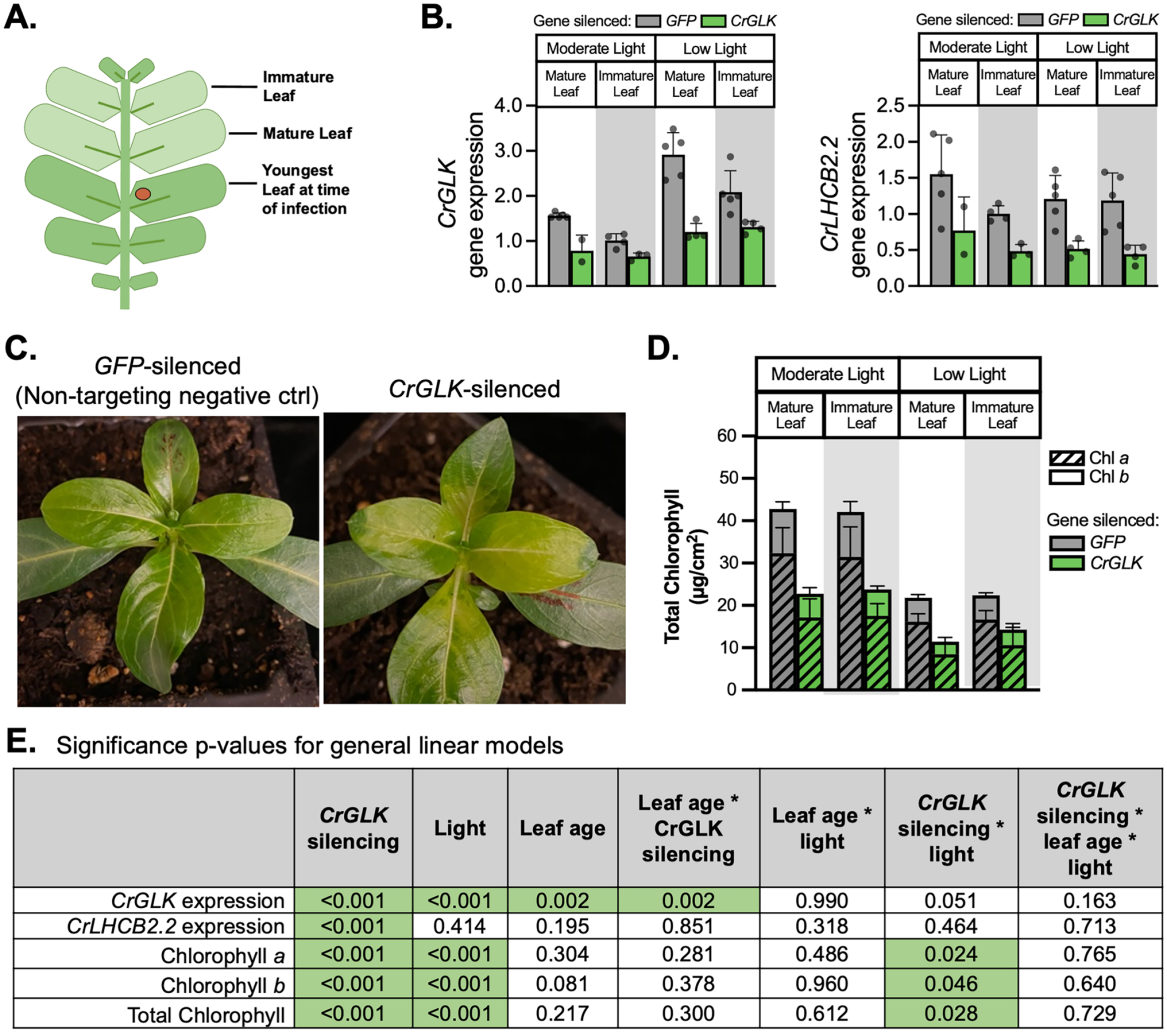
Silencing *CrGLK* decreased expression of *CrLHCB2.2* and chlorophyll content. **A** Setup of the VIGS experiment: plants were infected with *A. tumefaciens* containing a silencing plasmid. The youngest leaf pair at the time of infection was marked with a red dot. Plants were then grown under either moderate light or low light for 2–3 weeks. Silencing occurred in the two leaf pairs to emerge after infection (“immature leaf” or “mature leaf”), which were harvested for chlorophyll, alkaloid, or mRNA analysis. **B** *CrGLK* and *CrLHCB2.2* gene expression levels in *GFP*- and *CrGLK*-silenced plants. RNA was extracted from one mature leaf and one immature leaf for each plant. Gene expression was measured with qPCR and calculated using the 2^-∆∆Ct^ method (Livak & Schmittgen 2001) relative to immature leaves of the non-targeting negative control (*GFP*-silenced) grown under moderate light, and normalized to the housekeeping gene *SAND* (Pollier et al. 2014). Bar graphs represent the mean with error bars indicating the standard deviation. Each replicate is from an individual plant (*N* = 2–5). **C** *CrGLK*-silenced plants exhibited a visible lightening of leaves compared to *GFP*-silenced plants (negative non-targeting control). Representative photos are from plants grown under low light. **D** Silencing *CrGLK* decreased total chlorophyll content: chlorophyll a (Chl a) + chlorophyll b (Chl b). Chlorophyll was extracted from 6 mm leaf discs and calculated from A_645_ and A_663_ measurements. Bar graphs represent the mean with error bars indicating the standard deviation of Chl a or Chl b levels. Each replicate is from an individual plant (N = 5–6). **E** Significance in p-values for the influence of factors *CrGLK*-silencing, light, leaf age, and their interactions on *CrGLK* or *CrLHCB2.2* gene expression and chlorophyll. For each dependent variable, a full factorial linear model was fitted and p-values were determined from a Type III sum of squares F-test. All p-values less than 0.05 were highlighted green, indicating a significant contribution to the model. For example, silencing *CrGLK* significantly impacted *CrLHCB2.2* expression and chlorophyll accumulation whereas the interaction of *CrGLK* silencing and light only significantly impacted chlorophyll accumulation (colour figure online)

Due to the importance of development and light in regulating both *CrGLK* and the vindoline pathway, we additionally measured the expression of *CrGLK*, the vindoline pathway, and additional TIA pathway genes (*G10H, TDC, STR, and CS/HL1*) in leaves of two different developmental states (mature leaves and immature leaves, Fig. 3A) and in plants grown under two different light environments: moderate light (red and blue LED lights: ~ 90 µmol m^−2^ s^−1^) or low light (white, fluorescent lights: ~ 15 µmol m^−2^ s^−1^).

Consistent with literature, all TIA pathway genes that we measured had significantly higher expression in immature leaves compared to mature leaves (Fig. 2B, statistics in Fig. 5C). One exception was *D4H*, which exhibited higher expression in mature rather than immature leaves. Additionally, moderate light intensity significantly increased some vindoline pathway genes (*T3O, NMT, D4H, DAT*) and other TIA pathway genes (*G10H, TDC*), compared to low light (Fig. 2B, statistics in Fig. 5C). In general, TIA pathway genes were most highly expressed in immature leaves and moderate light.

In contrast to TIA pathway genes, *CrGLK* was most highly expressed in mature leaves and low light (Fig. 2B). This negative correlation between *CrGLK* and vindoline pathway gene expression in leaves of differing developmental states and light environments suggested that CrGLK may not be an activator of vindoline pathway gene expression. However, correlation of gene expression is only an initial clue pointing towards regulatory relationships. We next silenced *CrGLK* in *C. roseus* in order to functionally characterize its role in regulating vindoline biosynthesis.

### Silencing CrGLK with VIGS decreased CrLHCB2.2 expression and total chlorophyll levels

To functionally characterize CrGLK, we transiently silenced *CrGLK* in *C. roseus* leaves using virus-induced gene silencing (VIGS). Due to the importance of light in regulating both *CrGLK* and the vindoline pathway, VIGS plants were grown under two different light environments: moderate light (red and blue LED lights: ~ 90 µmol m^−2^ s^−1^) or low light (white, fluorescent lights: ~ 15 µmol m^−2^ s^−1^). The two leaf pairs to emerge after infection were harvested separately (i.e. mature leaves and immature leaves) to quantify chlorophyll, alkaloids, or gene expression (Fig. 3A). *CrGLK* gene expression was successfully silenced (> 40%, *p* < 0.001) in both mature and immature leaves and in both light environments compared to the non-targeting *GFP*-silenced control (Fig. 3B). The GFP-silenced control contained a 471 bp fragment in the pTRV2 silencing plasmid that did not target any native *C. roseus* genes.

Before exploring whether *CrGLK*-silencing impacted TIA biosynthesis, we validated our identification of *CrGLK* and VIGS methodology by examining well-characterized phenotypes of *GLK* knockout mutants: reduced chlorophyll accumulation and reduction of photosynthesis-associated nuclear genes (PhANGs) (Hills et al. 2015; K. Kobayashi et al. 2012; Koichi Kobayashi et al. 2013; Rauf et al. 2013; Waters et al. 2008, 2009; Zubo et al. 2018). As a representative PhANG and positive control, we identified and monitored the expression of a homologue of the Light Harvesting Complex Subunit B2.2 (*CrLHCB2.2*, CRO_T101917). *LHCB2.2* was previously characterized in *A. thaliana* as a gene strongly activated by AtGLK1 and AtGLK2 (Hills et al. 2015; Rauf et al. 2013; Waters et al. 2009). We observed decreases in *CrLHCB2.2* expression with *CrGLK-*silencing in all conditions (23–56%, *p* < 0.001), confirming the expected regulatory relationship between CrGLK and *CrLHCB2.2* (Fig. 3B).

We also examined the effect of *CrGLK* silencing on chlorophyll accumulation. It has been shown in many studies that GLKs positively regulate chlorophyll biosynthesis (K. Kobayashi et al. 2012; Koichi Kobayashi et al. 2013; Waters et al. 2008, 2009; Zubo et al. 2018). Our results are consistent with these studies; *CrGLK*-silenced plants exhibited a visible phenotype of light green leaves (Fig. 3C, Fig. S1) and significantly decreased levels of chlorophyll *a* and chlorophyll *b* (Fig. 3D). This phenotype is consistent with what was observed in *A. thaliana glk1glk2* double mutants but not in single-silenced plants (Fitter et al. 2002), confirming that *C. roseus* encodes only one GLK and that *CrGLK* is positively associated with chlorophyll accumulation. These results, the decrease in *CrLHCB2.2* expression and chlorophyll levels with *CrGLK*-silencing, confirmed that CrGLK shares not only structural homology but also functional homology with GLKs of other plant species. Importantly, these results also suggested that silencing *CrGLK* is sufficient to observe changes in gene expression and metabolite accumulation of the pathways that it regulates. We next determined the impact of *CrGLK-*silencing on TIA biosynthesis.

### Silencing CrGLK with VIGS increased TIA levels

Based on the correlation between the expression of the vindoline pathway in young, light exposed leaves and the activity of GLKs under these conditions in other plant species, we originally hypothesized that CrGLK might activate the vindoline pathway and increase TIA biosynthesis. This hypothesis was challenged when our results showed that *CrGLK* gene expression is negatively correlated with vindoline pathway gene expression in leaves of plants grown under different light intensities (Fig. 2B). Consistent with this negative correlation, we found that *CrGLK-*silencing led to an increase in TIA levels, suggesting that CrGLK may act as a repressor rather than an activator of TIA biosynthesis.

**Fig. 4:**
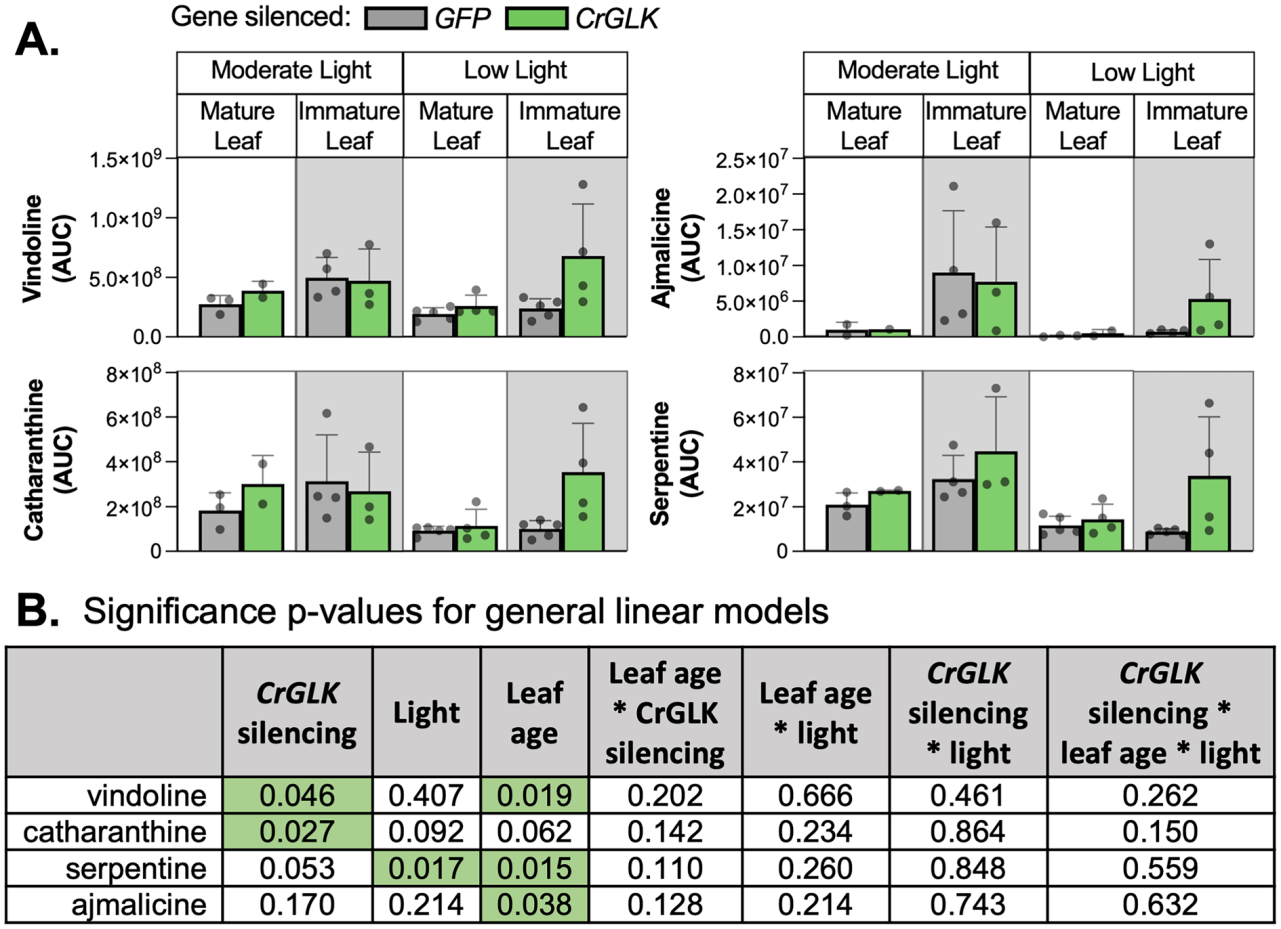
Silencing *CrGLK* increased TIAs (vindoline, catharanthine, ajmalicine, and serpentine) in immature leaves under low light. **A** Metabolite levels were estimated from the area under the curve (AUC) of the extracted ion chromatogram using HPLC–MS-MS with selected reaction monitoring. Bar graphs represent the mean with error bars indicating the standard deviation. Each replicate is from an individual plant (*N* = 2–5). (**B**) Significance p-values for the influence of factors *CrGLK*-silencing, light, and leaf age on alkaloid levels. For each dependent variable, a full factorial linear model was fitted and p-values were determined from a Type III sum of squares F-test. All p-values less than 0.05 were highlighted green, indicating a significant contribution to the model (colour figure online)

The effect of *CrGLK-*silencing on TIA levels was most prominent in immature leaves under low light where > twofold increases in vindoline, catharanthine, serpentine, and ajmalicine were observed (Fig. 4A; *p* < 0.05 for vindoline and catharanthine, Fig. 4B). In low light, basal *CrGLK* expression is high, and vindoline pathway gene expression is low (Fig. 2B). This may explain why we observed greater increases in TIA levels when *CrGLK* was silenced in low light compared to moderate light.

**Fig. 5:**
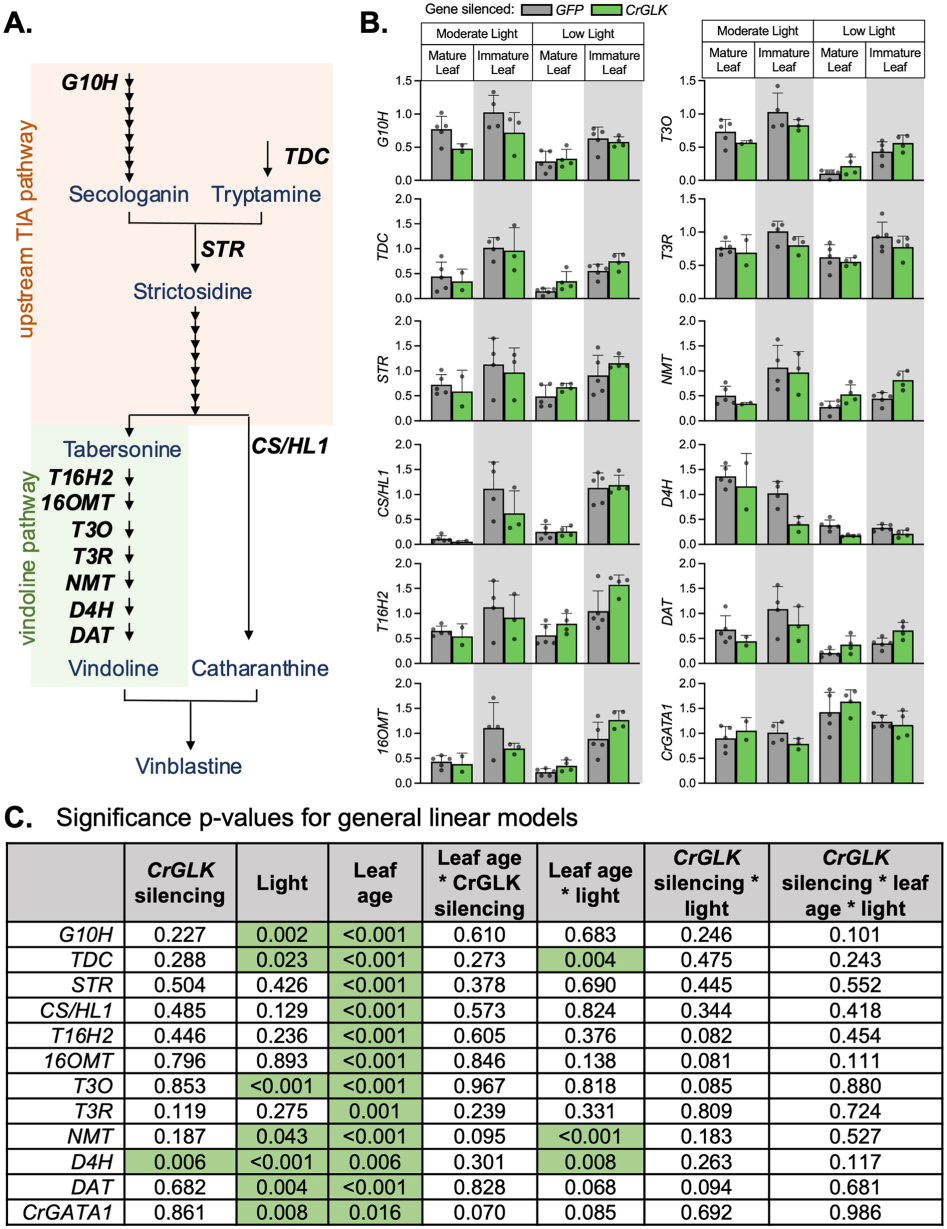
*CrGLK* silencing has little effect on TIA gene expression. **A** A simplified TIA biosynthetic pathway indicating enzymes whose gene expression was monitored in this experiment (the vindoline pathway: T16H2, 16OMT, T3O, T3R, NMT, D4H, DAT; upstream enzymes: G10H, TDC, STR; catharanthine synthase: CS/HL1). **B** Gene expression levels in *GFP*- and *CrGLK*-silenced plants. RNA was extracted from one mature leaf and one immature leaf for each plant (N = 2–5). Relative gene expression was measured with qPCR and calculated using the 2^-∆∆Ct^ method (Livak & Schmittgen 2001) relative to the non-targeting negative control condition (*GFP*-silenced plants) of immature leaves grown under moderate light, and normalized relative to the housekeeping gene, *SAND* (Pollier et al. 2014). Bar graphs represent the mean with error bars indicating the standard deviation. Each replicate is from an individual plant (N = 2–5). **C** Significance p-values for the influence of factors *CrGLK*-silencing, light, and leaf age on gene expression. For each gene, a full factorial linear model was fitted and p-values were determined from a Type III sum of squares F-test. All p-values less than 0.05 were highlighted green, indicating a significant contribution to the model (colour figure online)

To further explore whether *CrGLK* repressed TIA pathway genes, we monitored the gene expression of several key upstream and downstream TIA pathway enzymes in *CrGLK*-silenced plants: *G10H, TDC, STR, CS/HL1*, and the vindoline pathway (Fig. 5A). In general, patterns of TIA pathway gene expression were similar to patterns of alkaloid accumulation. The expression of most TIA pathway genes increased when *CrGLK* was silenced in low light (Fig. 5C). However, increases were small, mostly less than twofold, and not significant (Fig. 5D).

Interestingly, one vindoline pathway gene, *D4H*, behaved differently from the other TIA pathway genes, and significantly decreased in expression (by ~ 40%) when *CrGLK* was silenced in all conditions. *D4H* also differed from the rest of the vindoline pathway by being more highly expressed in mature leaves (Fig. 2B). These results were replicated in two additional experiments and with a second *CrGLK-*silencing fragment (Fig. S2). Consistently, we found that silencing *CrGLK* decreased expression of *D4H* while increasing expression of other vindoline pathway genes.

We additionally checked expression of *CrGATA1*, which encodes a previously-identified activator of the vindoline pathway (Yongliang Liu et al. 2019). GATAs and GLKs work together to coordinate chloroplast development in other plant species (Bastakis et al. 2018; Ohnishi et al. 2018; Zubo et al. 2018), so we speculated that *CrGATA1* expression may have increased in response to *CrGLK* silencing as a compensatory mechanism. Since CrGATA1 activates the vindoline pathway, this could explain the increases in vindoline observed with *CrGLK*-silencing. However, we did not observe any changes in *CrGATA1* gene expression when *CrGLK* was silenced, indicating that the effects of *CrGLK-*silencing on TIA accumulation were independent of CrGATA1.

Overall, silencing *CrGLK* with VIGS led to significantly decreased chlorophyll content (p < 0.001), *CrLHCB2.2* expression (p < 0.001), and *D4H* expression (p < 0.006) and increased vindoline and catharanthine content (p < 0.05).

### Reduced CrGLK expression with chloroplast retrograde signaling inducers increased TIA gene expression

VIGS is often variable and in our experiment *CrGLK* expression was only silenced by 50%. This may have contributed to the small, variable changes in TIA and gene expression levels with *CrGLK-*silencing. Using a similar approach, we studied the effect of reduced *CrGLK* expression on TIA biosynthesis using chemicals that induce chloroplast retrograde signaling. *GLK* gene expression is known to be strongly regulated by chloroplast retrograde signaling in other species (Hills et al. 2015; Kakizaki et al. 2009; Leister & Kleine 2016; Martin et al. 2016; Tokumaru et al. 2017; Waters et al. 2009). Two chemicals, norflurazon (Nor) and lincomycin (Lin), are known to induce retrograde signaling, repress *GLK* and *LHCB2.2* expression, and disrupt chloroplast development (Hills et al. 2015; Kakizaki et al. 2009; Leister and Kleine 2016; Martin et al. 2016; Waters et al. 2009). The mechanism of action by these chemicals differs; Nor disrupts carotenoid synthesis (Brausemann et al. 2017; Qin et al. 2007) while Lin disrupts translation within plastids (Mulo et al. 2003; D. N. Wilson 2014; S. B. Wilson & Moore 1973). To reduce *CrGLK* expression, we germinated *C. roseus* seedlings in the presence of these two chemicals and studied their effect on the expression of TIA pathway genes.

**Fig. 6:**
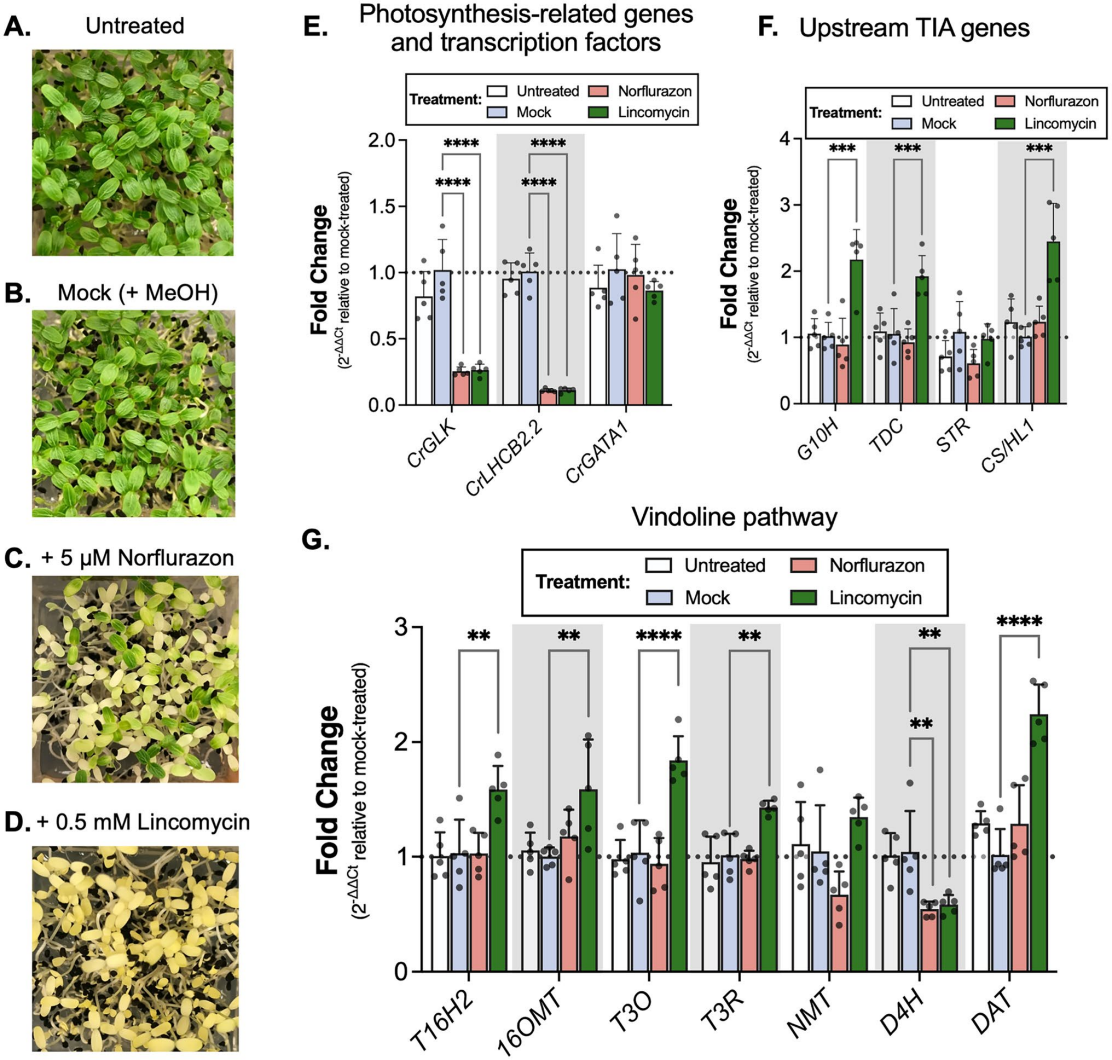
Lincomycin repressed the expression of *CrGLK* and *CrLHCB2.2* and activated the expression of most vindoline pathway genes. **A**–**D** Seeds were spread on Gamborg’s B5 media (without sucrose) containing 5 µM norflurazon (Nor) or 0.5 mM lincomycin (Lin). Nor was added from a stock solution dissolved in methanol (MeOH) while Lin was added from a stock solution dissolved in water. Mock-treated seedlings were spread on media containing an equal volume of MeOH as was added to Nor-treated seedlings. Compared to untreated and mock-treated seedlings, seedlings germinated in the presence of Nor or Lin showed inhibited chloroplast development after two days in the light. **E** *CrGLK* and *CrLHCB2.2* expression was significantly inhibited by both Nor and Lin while *CrGATA1* expression was unaffected. **F** *G10H, TDC, *and *CS/HL1* expression increased with Lin treatment while *STR* expression was unaffected. **G** Many vindoline pathway genes increased in expression under Lin treatment, except for *D4H*, which decreased in expression under both Nor and Lin treatments. Relative gene expression was measured with qPCR and calculated using the 2^-∆∆Ct^ method (Livak & Schmittgen 2001) relative to the mock condition, and normalized relative to the housekeeping gene, *SAND *(Pollier et al. 2014). ****p < 0.0001, ***p < 0.001 **p < 0.01, *p < 0.05 one-way ANOVA (adjusted for FDR) followed by a Dunnett’s test. Bar graphs represent the mean with error bars indicating the standard deviation. Each replicate is a pool of 5 seedlings (*N* = 5 replicates)

Like *A. thaliana* and other species (Oelmüller & Mohr 1986), *C. roseus* seedlings treated with Nor or Lin showed normal photomorphogenic development when exposed to light (i.e., opened cotyledons and shortened hypocotyls) but were completely white/yellow, indicating disrupted chloroplast development (Fig. 6A-D). As expected, both treatments caused significant decreases in the expression of *CrGLK* and *CrLHCB2.2* (~ 75% and 90%, respectively) while *CrGATA1* expression was unaffected (Fig. 6E).

The expression of most vindoline pathway genes increased with Lin treatment (significant increases in *T16H2*, *16OMT*, *T3O*, *T3R*, and *DAT* of 50 – 125%, Fig. 6G), similar to their increased expression with *CrGLK-*silencing (Fig. 5B, immature leaves under low light). In addition, the expression of *D4H* significantly decreased (~ 40%, Fig. 6G), as observed with *CrGLK-*silencing via VIGS (Fig. 5B). The expression of the upstream TIA pathway genes also significantly increased with Lin treatment (> twofold increase in *G10H*, *TDC*, CS/*HL1*; Fig. 6F). Interestingly and unexpectedly, Nor treatment did not have the same effect on the expression of the upstream and downstream TIA pathway or vindoline pathway genes (Fig. 6F). Thus reduced *CrGLK* expression alone was not sufficient to activate the expression of TIA pathway genes. These results were replicated and reproducible with Nor and Lin in the presence of 1% sucrose (Fig. S3).

As additional evidence, we attempted to transiently overexpress *CrGLK* in wild-type *C. roseus* seedlings grown under moderate light using our efficient *Agro*-mediated seedling infiltration (EASI) method. However, we did not see an increase in *CrLHCB2.2* gene expression (Fig. S4), suggesting that the overexpressed *CrGLK* was potentially degraded or inactive.

## Discussion

Terpenoid indole alkaloid (TIA) biosynthesis in *Catharanthus roseus* leaves is strongly dependent on the developmental state, with higher gene expression in immature leaves ((Besseau et al. 2013; Góngora-Castillo et al. 2012; Mall et al. 2019; Qu et al. 2015; B St-Pierre et al. 1999; Benoit St-Pierre et al. 1998), Fig. 2). However, the molecular mechanisms responsible for this developmental regulation are not known. In this paper, we investigated the role of Golden2-like (GLK) transcription factors in the developmental activation of TIA biosynthesis, particularly vindoline biosynthesis, as GLKs positively regulate chloroplast biogenesis and repress senescence in young leaves (Garapati et al. 2015; K. Kobayashi et al. 2012; Koichi Kobayashi et al. 2013; Rauf et al. 2013; Waters et al. 2008, 2009; Zubo et al. 2018). In silico investigations supported this hypothesis (Fig. 2A); for example, *CrGLK* and TIA pathway genes, particularly the vindoline pathway genes, are strongly expressed in immature leaves (Fig. 2A). We also identified multiple potential GLK binding sites in the vindoline pathway promoters (Table S4). But silencing or reducing *CrGLK* expression using virus-induced gene silencing or chloroplast retrograde signaling inducers instead showed a negative correlation between *CrGLK* expression and TIA biosynthesis (Fig. 4, 5, and 6). Our most important findings are discussed below.

GLKs are duplicated in many but not all species (Fitter et al. 2002; P. Wang et al. 2013). We identified only a single GLK homologue in *C. roseus* that contained the conserved GARP domain, GCT box, and “AREAEAA” motif (Fig. 1). Similar to phenotypes observed in double-knockout *glk1glk2* mutants in other species, silencing *CrGLK* in *C. roseus* leaves was sufficient to reduce chlorophyll levels and decrease expression of a chloroplast-associated gene, *LHCB2.2*, confirming the function and identification of a single *CrGLK* gene (Fig. 3).

We investigated the influence of light intensity and developmental state on the expression of *CrGLK* and the TIA pathway, particularly the vindoline pathway. Previous studies on the light-associated regulation of the vindoline pathway have mostly been investigated in light compared to dark rather than with varying light intensities. In *A. thaliana*, *GLK*s are transcriptionally repressed by PIFs in the dark and transcriptionally activated in the light (Fitter et al. 2002; Martin et al. 2016; Song et al. 2014), similar to the vindoline pathway (Yongliang Liu et al. 2019; Schröder et al. 1999; Vazquez-Flota and De Luca 1998; Yu et al. 2018). However, as light intensity increased, *GLK* expression was repressed, highlighting that its regulation by light was not binary ((Martin et al. 2016), Fig. 2B). With increasing light intensity, we observed that the expression of many TIA pathway genes increased, in contrast to *CrGLK* expression which decreased. This was the first clue suggesting that CrGLK may not activate the vindoline pathway.

Due to the low efficiency of developing transgenic *C. roseus* plants, the role of CrGLK in regulating the TIA and vindoline pathways was evaluated by reducing *CrGLK* expression using virus-induced gene silencing (VIGS) or using the chloroplast retrograde signaling inducers lincomycin and norflurazon. Silencing *CrGLK* by VIGS led to increased TIA accumulation and gene expression (Fig. 4 and 5), suggesting that CrGLK may repress, rather than activate, TIA biosynthesis. This effect was most prominent in immature leaves grown under low light when *CrGLK* expression is normally high.

The effects with lincomycin treatment were highly consistent with *CrGLK*-silencing experiments; while *CrGLK* and *CrLHCB2.2* were strongly repressed, vindoline pathway genes, along with other TIA genes (*G10H, TDC,* and *CS/HL1*), were significantly induced. Both VIGS and chloroplast retrograde signaling experiments showed that CrGLK does not activate but instead might repress TIA biosynthesis. Although we overexpressed *CrGLK* in wild-type *C. roseus* seedlings, we did not observe activation of *CrLHCB2.2*, suggesting that the overexpressed CrGLK was inactive. Thus, we were unable to further probe the mechanism of how CrGLK regulates TIA biosynthesis in this study (for example, by using promoters with native versus mutated GLK-binding motifs). Whether this activation was caused by direct binding of CrGLK to TIA pathway promoters or was an indirect effect of another factor regulated by CrGLK remains to be explored.

Interestingly, norflurazon treatment starkly differed from lincomycin treatment. Although *CrGLK* and *CrLHCB2.2* expression were repressed, norflurazon had no effect on the expression of TIA pathway genes. It is unclear why these two chemicals differed in their effect on TIA biosynthesis, but this experiment showed that reducing CrGLK expression alone is not sufficient to increase TIA pathway expression and that other mechanisms are involved. The role of chloroplast retrograde signaling in regulating TIA biosynthesis has not been explored previously.

Throughout these investigations, the regulation of *D4H* emerged as an outlier within the vindoline pathway. Our results indicated that CrGLK may activate *D4H* expression while antagonizing TIA biosynthesis in general. *D4H* expression was consistently repressed when *CrGLK* level was reduced with VIGS, lincomycin, or norflurazon treatment. Unfortunately, we were unable to explore this relationship directly as the transient overexpression of *CrGLK* did not induce the expression of our positive control, *CrLHCB2.2* (Fig. S4). Future experiments will be needed to determine if GLK directly binds and activates the *D4H* promoter or whether these were indirect effects caused by another factor.

## Conclusions

In this study, we identified and functionally characterized a single GLK homologue in the important medicinal plant *C. roseus.* We showed that *CrGLK* expression was positively associated with chlorophyll accumulation, *CrLHCB2.2* expression, and *D4H* expression, but was negatively associated with vindoline and catharanthine content and TIA pathway gene expression. This conclusion was supported by two different methods for reducing *CrGLK* expression (i.e. virus-induced gene silencing and application of chloroplast retrograde signaling inducer lincomycin). In addition to the role of *CrGLK* in regulating TIA biosynthesis, our paper is the first to show that chloroplast retrograde signaling can influence TIA biosynthesis. Understanding the mechanisms underlying how chloroplast retrograde signaling influences TIA biosynthesis and other defense-associated specialized metabolism is an exciting area of future inquiry.

### Supplementary Information

Below is the link to the electronic supplementary material.

Supplementary file1 (DOCX 7681 KB)

## Data Availability

Vindoline pathway promoter and 5’UTR sequences were deposited in Genbank (Accessions: OR052132-OR052138). L0 and pTRV2 plasmids were deposited at Addgene (IDs: 203,886 – 203,895; 203,903 – 203,906). Datasets generated during the current study are available from the corresponding author on reasonable request.

## References

[CR1] Aerts RJ, Gisi D, De Carolis E, De Luca V, Baumann TW (1994). Methyl jasmonate vapor increases the developmentally controlled synthesis of alkaloids in *Catharanthus* and *Cinchona* seedlings. Plant J.

[CR2] Alem AL, Ariel FD, Cho Y, Hong JC, Gonzalez DH, Viola IL (2022). TCP15 interacts with GOLDEN2-LIKE 1 to control cotyledon opening in *Arabidopsis*. Plant J.

[CR3] Balsevich J, de Luca V, Kurz WG (1986). Altered alkaloid pattern in dark grown seedlings of *Catharanthus roseus*. the isolation and characterization of 4-desacetoxyvindoline: a novel indole alkaloid and proposed precursor of vindoline. Heterocycles.

[CR4] Bastakis E, Hedtke B, Klermund C, Grimm B, Schwechheimer C (2018). LLM-domain B-GATA transcription factors play multifaceted roles in controlling greening in *Arabidopsis*. Plant Cell.

[CR5] Besseau S, Kellner F, Lanoue A, Thamm AMK, Salim V, Schneider B, Courdavault V (2013). A pair of tabersonine 16-hydroxylases initiates the synthesis of vindoline in an organ-dependent manner in *Catharanthus roseus*. Plant Physiol.

[CR6] Bolger AM, Lohse M, Usadel B (2014). Trimmomatic: a flexible trimmer for Illumina sequence data. Bioinformatics.

[CR7] Brausemann A, Gemmecker S, Koschmieder J, Ghisla S, Beyer P, Einsle O (2017). Structure of phytoene desaturase provides insights into herbicide binding and reaction mechanisms involved in carotene desaturation. Structure.

[CR8] Bravo-Garcia A, Yasumura Y, Langdale JA (2009). Specialization of the Golden2-like regulatory pathway during land plant evolution. New Phytol.

[CR9] Brown PD, Tokuhisa JG, Reichelt M, Gershenzon J (2003). Variation of glucosinolate accumulation among different organs and developmental stages of *Arabidopsis thaliana*. Phytochemistry.

[CR10] Brütting C, Schäfer M, Vanková R, Gase K, Baldwin IT, Meldau S (2017). Changes in cytokinins are sufficient to alter developmental patterns of defense metabolites in *Nicotiana attenuata*. Plant J : Cell Mol Biol.

[CR11] Colinas M, Pollier J, Vaneechoutte D, Malat DG, Schweizer F, De Milde L, Goossens A (2021). Subfunctionalization of paralog transcription factors contributes to regulation of alkaloid pathway branch choice in *Catharanthus roseus*. Front Plant Sci.

[CR12] DeLuca V, Balsevich J, Tyler RT, Eilert U, Panchuck BD, Kurz WGW (1986). Biosynthesis of Indole alkaloids: developmental regulation of the biosynthetic pathway from tabersonine to vindoline. J Plant Physiol.

[CR13] Engler C, Youles M, Gruetzner R, Ehnert TM, Werner S, Jones JDG, Marillonnet S (2014). A Golden Gate modular cloning toolbox for plants. ACS Synth Biol.

[CR14] Fernandez-Pozo N, Rosli HG, Martin GB, Mueller LA (2015). The SGN VIGS tool: user-friendly software to design virus-induced gene silencing (VIGS) constructs for functional genomics. Mol Plant.

[CR15] Fitter DW, Martin DJ, Copley MJ, Scotland RW, Langdale JA (2002). *GLK* gene pairs regulate chloroplast development in diverse plant species. Plant J.

[CR16] Franke J, Kim J, Hamilton JP, Zhao D, Pham GM, Wiegert-Rininger K, O’Connor SE (2019). Gene discovery in Gelsemium highlights conserved gene clusters in monoterpene indole alkaloid biosynthesis. ChemBioChem.

[CR17] Garapati P, Xue G-P, Munné-Bosch S, Balazadeh S (2015). Transcription factor ATAF1 in arabidopsis promotes senescence by direct regulation of key chloroplast maintenance and senescence transcriptional cascades. Plant Physiol.

[CR18] Gershenzon J, Ullah C (2022). Plants protect themselves from herbivores by optimizing the distribution of chemical defenses. Proc Natl Acad Sci.

[CR19] Gleadow RM, Woodrow IE (2000). Temporal and spatial variation in cyanogenic glycosides in *Eucalyptus cladocalyx*. Tree Physiol.

[CR20] Goklany S, Loring RH, Glick J, Lee-Parsons CWT (2009). Assessing the limitations to terpenoid indole alkaloid biosynthesis in *Catharanthus roseus* hairy root cultures through gene expression profiling and precursor feeding. Biotechnol Prog.

[CR21] Góngora-Castillo E, Childs KL, Fedewa G, Hamilton JP, Liscombe DK, Magallanes-Lundback M, Buell CR (2012). Development of transcriptomic resources for interrogating the biosynthesis of monoterpene indole alkaloids in medicinal plant species. PLoS ONE.

[CR22] Han X-Y, Li P-X, Zou L-J, Tan W, Zheng T, Zhang D-W, Lin H-H (2016). GOLDEN2-LIKE transcription factors coordinate the tolerance to Cucumber mosaic virus in *Arabidopsis*. Biochem Biophys Res Commun.

[CR23] Han Y, Li F, Wu Y, Wang D, Luo G, Wang X, Larkin RM (2024). PSEUDO-ETIOLATION IN LIGHT proteins reduce greening by binding GLK transcription factors. Plant Physiol.

[CR24] Hernández-Domínguez E, Campos-Tamayo F, Vázquez-Flota F (2004). Vindoline synthesis in in vitro shoot cultures of *Catharanthus roseus*. Biotech Lett.

[CR25] Hills AC, Khan S, López-Juez E (2015). Chloroplast biogenesis-associated nuclear genes: control by plastid signals evolved prior to their regulation as part of photomorphogenesis. Front Plant Sci.

[CR26] Kachroo P, Burch-Smith TM, Grant M (2021). An emerging role for chloroplasts in disease and defense. Annu Rev Phytopathol.

[CR27] Kakizaki T, Matsumura H, Nakayama K, Che F-S, Terauchi R, Inaba T (2009). Coordination of plastid protein import and nuclear gene expression by plastid-to-nucleus retrograde signaling. Plant Physiol.

[CR28] Kim D, Langmead B, Salzberg SL (2015). HISAT: a fast spliced aligner with low memory requirements. Nat Methods.

[CR29] Kobayashi K, Baba S, Obayashi T, Sato M, Toyooka K, Keranen M, Masuda T (2012). Regulation of root greening by light and auxin/cytokinin signaling in *Arabidopsis*. Plant Cell.

[CR30] Kobayashi K, Sasaki D, Noguchi K, Fujinuma D, Komatsu H, Kobayashi M, Masuda T (2013). Photosynthesis of root chloroplasts developed in *Arabidopsis* lines overexpressing GOLDEN2-LIKE transcription factors. Plant Cell Physiol.

[CR31] Leister D, Kleine T (2016). Definition of a core module for the nuclear retrograde response to altered organellar gene expression identifies GLK overexpressors as gun mutants. Physiol Plant.

[CR32] Liscombe DK, O’Connor SE (2011). A virus-induced gene silencing approach to understanding alkaloid metabolism in *Catharanthus roseus*. Phytochemistry.

[CR33] Liscombe DK, Usera AR, O’Connor SE (2010). Homolog of tocopherol C methyltransferases catalyzes N methylation in anticancer alkaloid biosynthesis. Proc Natl Acad Sci.

[CR34] Liu Y, Schiff M, Dinesh-Kumar SP (2002). Virus-induced gene silencing in tomato. Plant J.

[CR35] Liu Y, Patra B, Pattanaik S, Wang Y, Yuan L (2019). GATA and phytochrome interacting factor transcription factors regulate light-induced vindoline biosynthesis in *Catharanthus roseus*. Plant Physiol.

[CR36] Livak KJ, Schmittgen TD (2001). Analysis of relative gene expression data using real-time quantitative PCR and the 2-ΔΔCT method. Methods.

[CR37] Makino S, Kiba T, Imamura A, Hanaki N, Nakamura A, Suzuki T, Mizuno T (2000). Genes encoding pseudo-response regulators: insight into his-to-asp phosphorelay and circadian rhythm in *Arabidopsis thaliana*. Plant Cell Physiol.

[CR38] Mall M, Verma RK, Gupta MM, Shasany AK, Khanuja SPS, Shukla AK (2019). Influence of seasonal and ontogenic parameters on the pattern of key terpenoid indole alkaloids biosynthesized in the leaves of *Catharanthus roseus*. S Afr J Bot.

[CR39] Martin G, Leivar P, Ludevid D, Tepperman JM, Quail PH, Monte E (2016). Phytochrome and retrograde signalling pathways converge to antagonistically regulate a light-induced transcriptional network. Nat Commun.

[CR40] Meldau S, Erb M, Baldwin IT (2012). Defence on demand: mechanisms behind optimal defence patterns. Ann Bot.

[CR41] Menke FLH, Champion A, Kijne JW, Memelink J (1999). A novel jasmonate- and elicitor- responsive element in the periwinkle secondary metabolite biosynthetic gene Str interacts with a jasmonate- and elicitor- inducible AP2- domain transcription factor, ORCA2. EMBO J.

[CR42] Mizukami H, Nordlöv H, Lee SL, Scott AI (1979). Purification and properties of strictosidine synthetase (an enzyme condensing tryptamine and secologanin) from *Catharanthus roseus* cultured cells. Biochemistry.

[CR43] Mortensen S, Bernal-Franco D, Cole LF, Sathitloetsakun S, Cram EJ, Lee-Parsons CWT (2019). EASI transformation: an efficient transient expression method for analyzing gene function in *Catharanthus roseus* seedlings. Front Plant Sci.

[CR44] Mulo P, Pursiheimo S, Hou C-X, Tyystjärvi T, Aro E-M (2003). Multiple effects of antibiotics on chloroplast and nuclear gene expression. Funct Plant Biol.

[CR45] Murmu J, Wilton M, Allard G, Pandeya R, Desveaux D, Singh J, Subramaniam R (2014). *Arabidopsis* GOLDEN2-LIKE (GLK) transcription factors activate jasmonic acid (JA)-dependent disease susceptibility to the biotrophic pathogen *Hyaloperonospora arabidopsidis*, as well as JA-independent plant immunity against the necrotrophic pa. Mol Plant Pathol.

[CR46] Naaranlahti T, Auriola S, Lapinjoki SP (1991). Growth-related dimerization of vindoline and catharanthine in *Catharanthus roseus* and effect of wounding on the process. Phytochemistry.

[CR47] Ochoa-López S, Villamil N, Zedillo-Avelleyra P, Boege K (2015). Plant defence as a complex and changing phenotype throughout ontogeny. Ann Bot.

[CR48] Oelmüller R, Mohr H (1986). Photooxidative destruction of chloroplasts and its consequences for expression of nuclear genes. Planta.

[CR49] Ohnishi A, Wada H, Kobayashi K (2018). Improved photosynthesis in *Arabidopsis* roots by activation of GATA transcription factors. Photosynthetica.

[CR50] Ördög V, Zoltán M (1949). Copper enzymes in isolated chloroplasts. Polyphenoloxidase in Beta Vulgaris Plant Physiol.

[CR51] Papazian S, Girdwood T, Wessels BA, Poelman EH, Dicke M, Moritz T, Albrectsen BR (2019). Leaf metabolic signatures induced by real and simulated herbivory in black mustard (*Brassica nigra*). Metabol : off J Metabol Soc.

[CR52] Paul P, Singh SK, Patra B, Sui X, Pattanaik S, Yuan L (2017). A differentially regulated AP2/ERF transcription factor gene cluster acts downstream of a MAP kinase cascade to modulate terpenoid indole alkaloid biosynthesis in *Catharanthus roseus*. New Phytol.

[CR53] Pertea M, Pertea GM, Antonescu CM, Chang T-C, Mendell JT, Salzberg SL (2015). StringTie enables improved reconstruction of a transcriptome from RNA-seq reads. Nat Biotechnol.

[CR54] Pollier J, Vanden Bossche R, Rischer H, Goossens A (2014). Selection and validation of reference genes for transcript normalization in gene expression studies in Catharanthus roseus. Plant Physiol Biochem.

[CR55] Qin G, Gu H, Ma L, Peng Y, Deng XW, Chen Z, Qu L-J (2007). Disruption of phytoene desaturase gene results in albino and dwarf phenotypes in Arabidopsis by impairing chlorophyll, carotenoid, and gibberellin biosynthesis. Cell Res.

[CR56] Qu Y, Easson MLAE, Froese J, Simionescu R, Hudlicky T, De Luca V (2015). Completion of the seven-step pathway from tabersonine to the anticancer drug precursor vindoline and its assembly in yeast. Proc Natl Acad Sci USA.

[CR57] Raina SK, Wankhede DP, Jaggi M, Singh P, Jalmi SK, Raghuram B, Sinha AK (2012). CrMPK3, a mitogen activated protein kinase from *Catharanthus roseus* and its possible role in stress induced biosynthesis of monoterpenoid indole alkaloids. BMC Plant Biol.

[CR58] Rauf M, Arif M, Dortay H, Matallana-Ramírez LP, Waters MT, Gil Nam H, Balazadeh S (2013). ORE1 balances leaf senescence against maintenance by antagonizing G2-like-mediated transcription. EMBO Rep.

[CR59] Rizvi NF, Weaver JD, Cram EJ, Lee-Parsons CWT (2016). Silencing the transcriptional repressor, ZCT1, illustrates the tight regulation of terpenoid indole alkaloid biosynthesis in *Catharanthus roseus* hairy roots. PLoS ONE.

[CR60] Savitch LV, Subramaniam R, Allard GC, Singh J (2007). The GLK1 “regulon” encodes disease defense related proteins and confers resistance to *Fusarium graminearum* in *Arabidopsis*. Biochem Biophys Res Commun.

[CR61] Schröder G, Unterbusch E, Kaltenbach M, Strack D, Luca VD, Schro J (1999). Light-induced cytochrome P450-dependent enzyme in indole alkaloid biosynthesis: tabersonine 16-hydroxylase. Fed Euro Biochem Soc.

[CR62] Schweizer F, Colinas M, Pollier J, Van Moerkercke A, Vanden Bossche R, de Clercq R, Goossens A (2018). An engineered combinatorial module of transcription factors boosts production of monoterpenoid indole alkaloids in *Catharanthus roseus*. Metab Eng.

[CR63] Singh SK, Patra B, Paul P, Liu Y, Pattanaik S, Yuan L (2020). Revisiting the ORCA gene cluster that regulates terpenoid indole alkaloid biosynthesis in *Catharanthus roseus*. Plant Sci.

[CR64] Singh SK, Patra B, Paul P, Liu Y, Pattanaik S, Yuan L (2021). *BHLH IRIDOID SYNTHESIS 3* is a member of a bHLH gene cluster regulating terpenoid indole alkaloid biosynthesis in *Catharanthus roseus*. Plant Direct.

[CR65] Song Y, Yang C, Gao S, Zhang W, Li L, Kuai B (2014). Age-triggered and dark-induced leaf senescence require the bHLH transcription factors PIF3, 4, and 5. Mol Plant.

[CR66] Stamp N (2003). Out of the quagmire of plant defense hypotheses. Q Rev Biol.

[CR67] St-Pierre B, Laflamme P, Alarco A, Luca E (1998). The terminal O-acetyltransferase involved in vindoline biosynthesis defines a new class of proteins responsible for coenzyme A-dependent acyl transfer. Plant J.

[CR68] St-Pierre B, Vazquez-Flota F, De Luca V, D. L.  (1999). Multicellular compartmentation of *Catharanthus roseus* alkaloid biosynthesis predicts intercellular translocation of a pathway intermediate. Plant Cell.

[CR69] Sun Z, Zhang K, Chen C, Wu Y, Tang Y, Georgiev MI, Zhou M (2018). Biosynthesis and regulation of cyanogenic glycoside production in forage plants. Appl Microbiol Biotech.

[CR70] Svec D, Tichopad A, Novosadova V, Pfaffl MW, Kubista M (2015). How good is a PCR efficiency estimate: Recommendations for precise and robust qPCR efficiency assessments. Biomol Detect Quantif.

[CR71] Tachibana R, Abe S, Marugami M, Yamagami A, Akema R, Ohashi T, Nakano T (2024). BPG4 regulates chloroplast development and homeostasis by suppressing GLK transcription factors and involving light and brassinosteroid signaling. Nat Commun.

[CR72] Tokumaru M, Adachi F, Toda M, Ito-Inaba Y, Yazu F, Hirosawa Y, Inaba T (2017). Ubiquitin-proteasome dependent regulation of the GOLDEN2-LIKE 1 transcription factor in response to plastid signals. Plant Physiol.

[CR73] Traw MB, Feeny P (2008). Glucosinolates and trichomes track tissue value in two sympatric mustards. Ecology.

[CR74] Tu X, Ren S, Shen W, Li J, Li Y, Li C, Zhong S (2022). Limited conservation in cross-species comparison of GLK transcription factor binding suggested wide-spread cistrome divergence. Nat Commun.

[CR75] van der Fits L, Memelink J (2000). ORCA3, a jasmonate-responsive transcriptional regulator of plant primary and secondary metabolism. Science.

[CR76] Van Moerkercke A, Steensma P, Schweizer F, Pollier J, Gariboldi I, Payne R, Goossens A (2015). The bHLH transcription factor BIS1 controls the iridoid branch of the monoterpenoid indole alkaloid pathway in *Catharanthus roseus*. Proc Nat Acad Sci United States Am.

[CR77] Van Moerkercke A, Steensma P, Gariboldi I, Espoz J, Purnama PC, Schweizer F, Goossens A (2016). The basic helix-loop-helix transcription factor BIS2 is essential for monoterpenoid indole alkaloid production in the medicinal plant *Catharanthus roseus*. Plant J.

[CR78] Vazquez-Flota FA, De Luca V (1998). Developmental and light regulation of desacetoxyvindoline 4-hydroxylase in Catharanthus roseus (L.) G. Don. Evidence of a multilevel regulatory mechanism. Plant Physiol.

[CR79] Vázquez-Flota FA, De Luca V (1998). Jasmonate modulates development- and light-regulated alkaloid biosynthesis in *Catharanthus roseus*. Phytochemistry.

[CR80] Wang Q, Yuan F, Pan Q, Li M, Wang G, Zhao J, Tang K (2010). Isolation and functional analysis of the *Catharanthus roseus* deacetylvindoline-4-O-acetyltransferase gene promoter. Plant Cell Rep.

[CR81] Wang P, Fouracre J, Kelly S, Karki S, Gowik U, Aubry S, Langdale JA (2013). Evolution of GOLDEN2-LIKE gene function in C3 and C4 plants. Planta.

[CR82] Waters MT, Moylan EC, Langdale JA (2008). GLK transcription factors regulate chloroplast development in a cell-autonomous manner. Plant J.

[CR83] Waters MT, Wang P, Korkaric M, Capper RG, Saunders NJ, Langdale JA (2009). GLK transcription factors coordinate expression of the photosynthetic apparatus in *Arabidopsis*. Plant Cell.

[CR84] Weber E, Engler C, Gruetzner R, Werner S, Marillonnet S (2011). A modular cloning system for standardized assembly of multigene constructs. PLoS ONE.

[CR85] Wei S (2010). Methyl jasmonic acid induced expression pattern of terpenoid indole alkaloid pathway genes in *Catharanthus roseus* seedlings. Plant Growth Regul.

[CR86] Wilson DN (2014). Ribosome-targeting antibiotics and mechanisms of bacterial resistance. Nat Rev Microbiol.

[CR87] Wilson SB, Moore AL (1973). The effects of protein synthesis inhibitors on oxidative phosphorylation by plant mitochondria. Biochimica Et Biophysica Acta (BBA)— Bioenergetics.

[CR88] Wingett SW, Andrews S (2018). FastQ screen: a tool for multi-genome mapping and quality control. F1000Research.

[CR89] Yu B, Liu Y, Pan Y, Liu J, Wang H, Tang Z (2018). Light enhanced the biosynthesis of terpenoid indole alkaloids to meet the opening of cotyledons in process of photomorphogenesis of Catharanthus roseus. Plant Growth Regul.

[CR90] Zhang D, Tan W, Yang F, Han Q, Deng X, Guo H, Lin H (2021). A BIN2-GLK1 signaling module integrates brassinosteroid and light signaling to repress chloroplast development in the dark. Develop Cell.

[CR91] Zhou P, Yang J, Zhu J, He S, Zhang W, Yu R, Huang X (2015). Effects of β-cyclodextrin and methyl jasmonate on the production of vindoline, catharanthine, and ajmalicine in *Catharanthus roseus* cambial meristematic cell cultures. Appl Microbiol Biotech.

[CR92] Zubo YO, Blakley IC, Franco-Zorrilla JM, Yamburenko MV, Solano R, Kieber JJ, Schaller GE (2018). Coordination of chloroplast development through the action of the GNC and GLK transcription factor families. Plant Physiol.

