# Supplementary material for: The role of the Golden2-like (GLK) transcription factor in regulating terpenoid indole alkaloid biosynthesis in Catharanthus roseus
Source: Plant Cell Rep. 2024 May 14;43(6):141. doi: 10.1007/s00299-024-03208-9 (PMC11093837; doi:10.1007/s00299-024-03208-9)
Supplement: Supplementary file 1 — Supplementary file1 (DOCX 7681 KB) [file 299_2024_3208_MOESM1_ESM.docx]

# The role of the GLK transcription factor in regulating terpenoid indole alkaloid biosynthesis in *Catharanthus roseus*

Lauren F. Cole-Osborn^1,2^, Shannon A. McCallan^3^, Olga Prifti^2^, Rafay Abu^3^, Virginie Sjoelund^3^, Carolyn W.T. Lee-Parsons^1,2,3,4^

^1^ Northeastern University, Department of Chemical Engineering, Boston, MA, USA 02115

^2^ Northeastern University, Department of Bioengineering

^3^ Northeastern University, Department of Chemistry and Chemical Biology

^4^ Corresponding author. ca.lee@northeastern.edu. ORCID = 0000-0001-5905-1214

## Supplementary Figures and Tables

**Table S1** Primers used for cloning in this study. All primers were originally designed. Uppercase indicates sequences complementary to its target while lowercase indicates 5’ overhangs to facilitate cloning

| **Primer Name** | **Primer Sequence** | **Purpose** | **Project** |
| --- | --- | --- | --- |
| GlkFattb1 | ggggacaagtttgtacaaaaaagcaggct GGTGGATTGGACACCAG | Amplification and cloning of *CrGLK* fragment for VIGS silencing | Virus-induced gene silencing of *CrGLK* |
| GlkRattb2 | ggggaccactttgtacaagaaagctgggt CCTCAGCTTCTCTTGCTAG |  |  |
| GLK_V2_F_attb1 | ggggacaagtttgtacaaaaaagcaggctCCCCAGGAACGCC | Amplification and cloning of *CrGLK* fragment 2 for VIGS silencing |  |
| GLK_V2_R_attb2 | ggggaccactttgtacaagaaagctgggtATAAAACATCTCCGATAGCTGC |  |  |
| *GFP*silenceATTB-F | ggggacaagtttgtacaaaaaagcaggctAAGAGTGCCATGCCCGAAGGTTAT | Amplification and cloning of *GFP* fragment for VIGS silencing |  |
| *GFP*silenceATTB-R | ggggaccactttgtacaagaaagctgggtAAGGGCAGATTGTGTGGACAGGTAA |  |  |
| CHLHFattb1 | ggggacaagtttgtacaaaaaagcaggctCAGTTGCCACACTAGTTAATATTGC | Amplification and cloning of *CHLH* fragment for VIGS silencing |  |
| CHLHRattb2 | ggggaccactttgtacaagaaagctgggtGCATGGATATTCTTTCCCGTTGGC |  |  |
| 16OMT_P_F1 | aaggtctcaacatggagTTATATTTGTTGCTGGCTATATATATAATAGTGGAC | Amplification and domestication of *16OMT* promoter | Amplification and sequencing of vindoline pathway promoter + 5’UTRs from *C. roseus* var. Little Bright Eye |
| 16OMT_P_R1 | aaggtctcaacaaTGTTCTTATATACAAAAAATATGACTTGGGAC |  |  |
| 16OMT_P_F2 | aaggtctcaacatAACACGAGACATGTGTAACTCAGTTG |  |  |
| 16OMT_P_R2 | aaggtctcaacaaATGGTATGGTATGCTATATATAGAGAGATGG |  |  |
| 16OMT_P_F3 | aaggtctcaacatCCATGTCTCTTCTCAAGAGAGCAC |  |  |
| 16OMT_P_R3 | aaggtctcaacaacattGGCGAGTTTCTGGGGG |  |  |
| T3O_P_F1_3 | aagaagacaaggagTATTATGGAGGTTAACAAATATTTACCGTAGATT | Amplification and domestication of *T3O* promoter |  |
| T3O_P_R1_3 | aagaagacaacattAATGAATTTTTTTTTGTTGGTTTGC |  |  |
| T3R_P_F1 | aaggtctcaacatggagCATGTATTGAACCTTAAATTTCAATAATCATC | Amplification and domestication of *T3R* promoter |  |
| T3R_P_R1 | aaggtctcaacaaACACCGAGATAAGGATAAGGTAAGGAAG |  |  |
| T3R_P_F2 | aaggtctcaacatGTGTCTCCTCTCTTCATATGTGG |  |  |
| T3R_P_R2 | aaggtctcaacaacattACATTAAGGAAAAGACAAGCAAAGC |  |  |
| T3R_P_F2 | aaggtctcaacatGTGTCTCCTCTCTTCATATGTGG |  |  |
| T3R_P_R2_2 | aaggtctcaacaacattTTCACTAGACATTAAGGAAAAGACAAGCAAAGC |  |  |
| NMT_P_F4 | aagaagacaaggagTGGCTCTGATACCACTGTTACG | Amplification and domestication of *NMT* promoter |  |
| NMT_P_R4 | aagaagacaacatTGTATTGTTGTATGTTTTGCTACCTCAC |  |  |
| NMT_P_F1 | aaggtctcaacatggagTGGCTCTGATACCACTGTTACG |  |  |
| NMT_P_R1 | aaggtctcaacaaCAGACCGATTGTAATAGATTAGATCTG |  |  |
| NMT_P_F2 | aaggtctcaacatTCTGCTTCCTTTCTTTCTTCCC |  |  |
| NMT_P_R2 | aaggtctcaacaaCTCTCGAGGTAAGGTTACAGCTTTC |  |  |
| NMT_P_F3 | aaggtctcaacatAGAGCTTTCAGGTCTGGGTCAG |  |  |
| NMT_P_R3 | aaggtctcaacaacattTGTATTGTTGTATGTTTTGCTACCTCAC |  |  |
| D4H_P_F1_2 | aagaagacaaggagTATTTCTTCTCACTAATAATAATTAATTTCATATTGTTC | Amplification and domestication of *D4H* promoter |  |
| D4H_P_R1_2 | aagaagacaacatTTTTCTTTCTTGCTCAGAATTTGG |  |  |
| DAT_P_F1 | aaggtctcaacatggagTGTTCAAACTATCAAATTCCAAGTG | Amplification and domestication of *DAT* promoter |  |
| DAT_P_R1_2 | aaggtctcaacaaCAAGACACATTTTGTAAGCAATATCC |  |  |
| DAT_P_F2_2 | aaggtctcaacatCTTGAATTAGAACCTTCCAATTCAG |  |  |
| DAT_P_R2 | aaggtctcaacaacattTTTGCTTGCTGTTATATATTCAGACC |  |  |
| GLK_ Primer1F | ttgaagacaaaATGCTTGCTGTGTCACCATTG | Amplification and domestication of *CrGLK* coding sequence | CrGLK overexpression |
| GLK_Primer1R | ttgaagacaaTGACTTCCTTCCTTTATCACCTTCT |  |  |
| GLK_Primer2F | ttgaagacaaGTCATCTTCTACTCAATCAAAGAATTCTTCTG | Amplification and domestication of *CrGLK* coding sequence |  |
| GLK_Primer2R | ttgaagacaaaagcTTACGTACAAACTGGTGGAATTTTC |  |  |
| LC31_GLK_F | aagaagacaaaATGCTTGCTGTGTCACCATTGAGGA | Cloning of *CrGLK* coding sequence without stop codon (to facilitate future C-terminal tags) |  |
| LC32_GLK_R | aagaagacaacgaaccCGTACAAACTGGTGGAATTTTCGGT |  |  |
| LC23_StopCodonF | aagaagacaattcgTAAgcttaagtcttcaa | Cloning of L0 (CT) plasmid containing a stop codon |  |
| LC24_StopCodonR | ttgaagacttaagcTTAcgaattgtcttctt |  |  |
| LC73_SDM_Ctrunc_F | TAAGCTTGGACTCCCATG | Site-directed mutagenesis of *CrGLK* overexpression plasmid to remove cloning artifacts |  |
| LC100_GLK_2AA_R | CGTACAAACTGGTGGAATTTTC |  |  |

**Table S2** Locations of introduced mutations for MoClo domestication

to remove BpiI and BsaI recognition sites. Location was calculated from the start codon for each gene (downstream is positive, upstream is negative). For vindoline pathway promoters, location is calculated according to the sequenced Little Bright Eye promoter rather than the predicted Sunstorm Apricot promoter. CDS = Coding Sequence; UTR = Untranslated Region

| **Sequence to domesticate** | **Location of mutation  (From start codon)** | **Basepair changed** |
| --- | --- | --- |
| *CrGLK* CDS | 474 | T > A |
| *16OMT* Promoter and 5’UTR | -607 | G > C |
| *16OMT* Promoter and 5’UTR | -37 | C > G |
| *T3R* Promoter and 5’UTR | -232 | C > G |
| *NMT* Promoter and 5’UTR | -742 | C > G |
| *NMT* Promoter and 5’UTR | -754 | C > G |
| *NMT* Promoter and 5’UTR | -853 | C > G |
| *DAT* Promoter and 5’UTR | -860 | C > G |

Table S3 qPCR primers used in this study. All primers were checked for efficiency and specificity before use

| **Primer Name** | **Primer Sequence** | **Purpose** | **Source** |
| --- | --- | --- | --- |
| GLK_qF3 | CGGTTCAGGCTCATCTACTTT | qPCR amplifying *CrGLK* | This study |
| GLK_qR3 | GACTTCCTTCCTTTATCACCTTCT |  |  |
| GNC_qF2 | GGAGGGACTAGCCAAGTAAAC | qPCR amplifying *CrGATA1* | This study |
| GNC_qR2 | CGGCTGCTGAAGTCATCTTA |  |  |
| q2T16h_up | GATCAACTCACAGTGGCAGTC | qPCR amplifying *T16H2* | This study |
| q2T16h_down | GACTTGAGACTTGTGATTGGC |  |  |
| 16OMTvigs-qF1 | GTGTGAAGATACTCAAAAGCTGC | qPCR amplifying *16OMT* | (David K Liscombe & O’Connor, 2011) |
| 16OMTvigs-qR1 | CAAAATTTACAAGCATTGCCATATCC |  |  |
| T3O_qF | GTCATAGACGAGCACAGAGAAA | qPCR amplifying *T3O* | This study |
| T3O_qR | CACCACCCTCTTCAATCCTAAG |  |  |
| T3R_qF | CTTGAGCCACTCTTTGCTTTAC | qPCR amplifying *T3R* | This study |
| T3R_qR | ATGAGGGACATTGCGGATAC |  |  |
| 2270-qF1 | TGACAAAGTAACCGGAGCATGGGA | qPCR amplifying *NMT* | (David K Liscombe & O’Connor, 2011) |
| 2270-qR1 | ATCCGAATGACGGCATCTTGGCTA |  |  |
| D4Hvigs-QF1 | TGGCCTCAGTAGCAATTCAG | qPCR amplifying *D4H* | (David K Liscombe & O’Connor, 2011) |
| D4Hvigs-QR1 | TCCATATTTCTCACTCGCTTCTC |  |  |
| DATvigs-qF1 | GAGGTTTTGACTGCTTTTCTCAG | qPCR amplifying *DAT* | (David K Liscombe & O’Connor, 2011) |
| DATvigs-qR1 | TGGAAATGGCAAAGATTGGC |  |  |
| SAND_qF | TGCTGTGGAGGAGGAAGAAG | qPCR amplifying *SAND* | (Pollier et al., 2014) |
| SAND_qR | ACTGGCGGAACTACTACTACC |  |  |
| LHCB2_qF | GTTGTTCTCATGGGCTTGATTG | qPCR amplifying *CrLHCB2.2* | This study |
| LHCB2_qR | AATGCTCCTCCTGGGTAGAT |  |  |
| G10h-forward | TAGCAGGGACGGACACAACATCAA | qPCR amplifying *G10H* | (Goklany et al., 2009) |
| G10h-reverse | TCACGTCCAATTGCCCAAGCATTC |  |  |
| Tdc forward | ACCTACGACCGTCGAAACGGATTT | qPCR amplifying *TDC* | (Goklany et al., 2009) |
| Tdc reverse | AAACTCGGGACATATACAGGCGCT |  |  |
| Str_F | GCTAGAAGGGCCAAAGAA | qPCR amplifying *STR* | (Rizvi et al., 2016) |
| Str_R | GGTGGTGGAAGTGGTATAA |  |  |
| HL1_qF | TGGGGCTGGCTTTTGTCTAGAATC | qPCR amplifying CS/*HL1* | (Qu et al., 2018) |
| HL1_qR | TAAGCTGCGGGTAAAAGGTGCTCT |  |  |

**Table S4** GLK core binding motifs (RGATTYY, where R = A or G and Y = C or T) (Tu et al., 2022) in vindoline pathway promoters and 5’UTRs (approximately 1 kb upstream of the start codon) in *C. roseus* var. Little Bright Eye (LBE: PCR-amplified and sequence-confirmed) or *C. roseus* var. Sunstorm Apricot (SA: extracted from *C. roseus* genome v. 2 (Franke et al., 2019). When the location differed between LBE and SA, both locations were given, with the LBE location indicated first. The Find Individual Motif Occurrences (FIMO) tool was used to find motifs and compute p-values from the log-odds score based on the default random model letter frequencies (A = 0.275, C = 0.225, G = 0.225, T=0.275) with a cutoff of 0.001

| **Promoter and 5’UTR** | **Motif** | **bp away from start codon** | **p-value** | **Cultivar** |
| --- | --- | --- | --- | --- |
| T16H2 | AGATTTT | 26 | 0.000583 | LBE |
|  | AGATTTC | 185 | 0.000487 | LBE |
| 16OMT | AGATTTC | 206 | 0.000487 | LBE and SA |
|  | GGATTGC | 224 | 0.000939 | LBE and SA |
|  | AGATTTT | 332/333 | 0.000583 | LBE and SA |
|  | AGATTCT | 534/535 | 0.000487 | LBE and SA |
|  | GGATTTC | 967/968 | 0.000249 | LBE and SA |
| T3O | GGATTTC | 348/269 | 0.000249 | LBE and SA |
|  | GGATTTC | 739/705 | 0.000249 | LBE and SA |
| T3R | AGATTTT | 118/110 | 0.000583 | LBE and SA |
| NMT | GGATTCT | 633/634 | 0.000249 | LBE and SA |
|  | AGATTCC | 910/911 | 0.000249 | LBE and SA |
| D4H | GGATTTT | 70 | 0.000487 | LBE and SA |
|  | GGATTAC | 81 | 0.000939 | LBE and SA |
|  | GGATTCC | 278 | 0.000054 | LBE |
|  | GGATTCT | 278 | 0.000249 | SA |
| DAT | AGATTTT | 372 | 0.000583 | LBE and SA |
|  | GGATTTT | 550 | 0.000487 | LBE and SA |


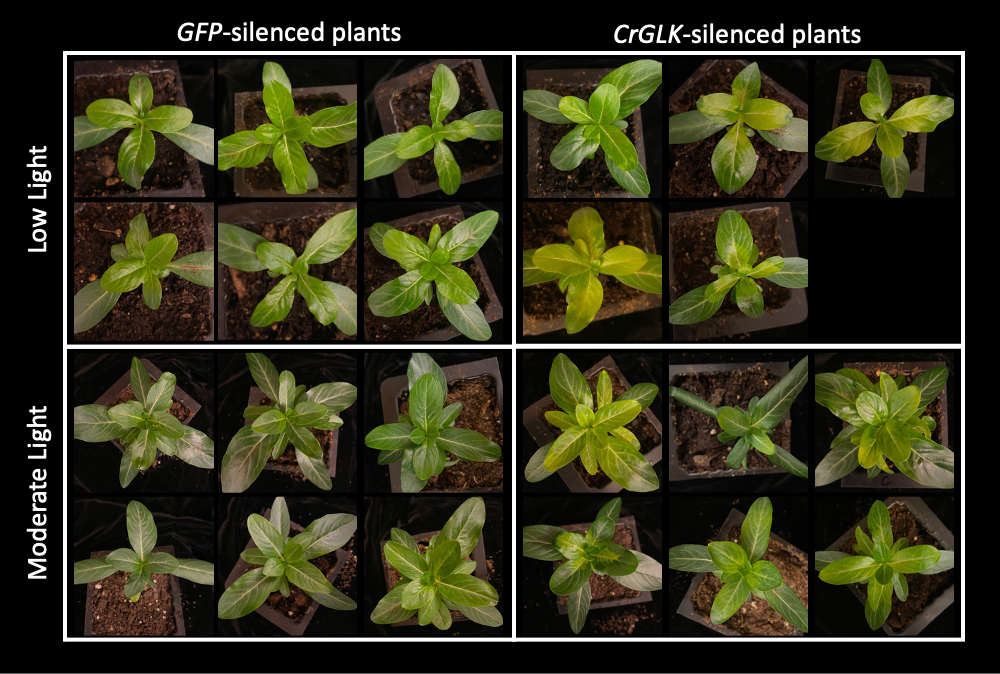


**Fig. S1** *CrGLK*-silenced plants showed lighter green leaves compared to *GFP*-silenced plants. A red dot indicated the youngest leaf at the time of infection. Plants were grown under moderate light (90 µmol m^-2^ s^-1^) and then transferred to either low light (15 µmol m^-2^ s^-1^) or moderate light (90 µmol m^-2^ s^-1^) for 3 weeks after infection. Leaves were harvested for chlorophyll quantification


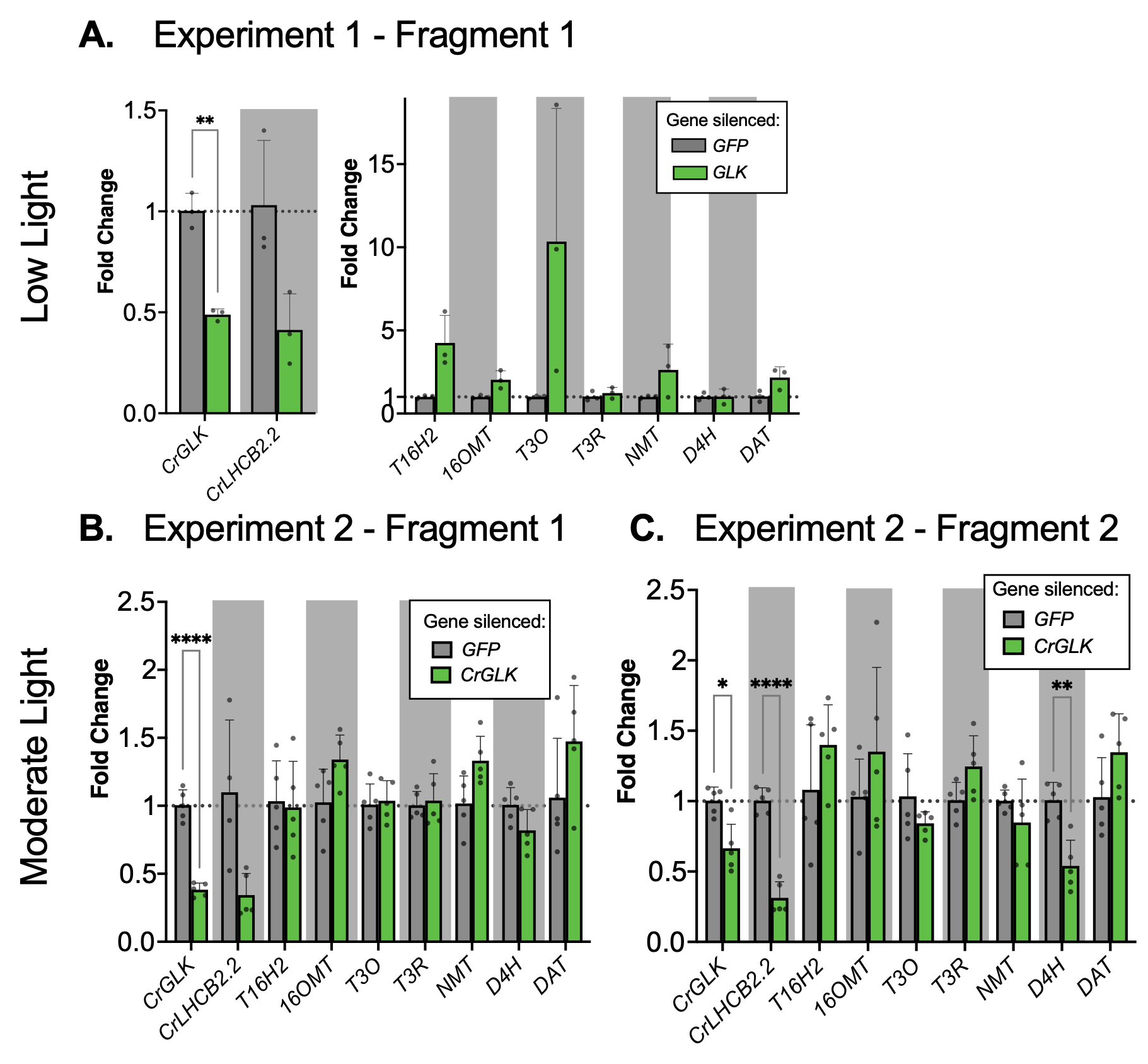


**Fig. S2** *CrGLK* silencing consistently showed enhanced expression of most vindoline pathway genes and decreased expression of *D4H* between replicate experiments and with different silencing fragments. (A) In Experiment 1, increases in vindoline pathway genes were observed when *CrGLK* was silenced with silencing fragment 1; plants were grown under low, white light (about 2000 Lux). Plants were grown in germination boxes with 1.4" square cells, planted every other cell. Prior to infection, plants were grown under red and blue LED lights (about 1150 Lux). Each replicate is one leaf from the immature leaf or second leaf pair after infection (N = 3 individual plants). (B-C) In Experiment 2, *CrGLK* was silenced in plants grown under moderate, red and blue light (90 µmol m^-2^ s^-1^) with either the first or second silencing fragment; non-significant increases in the expression of vindoline pathway genes and either significant or non-significant decreases in *D4H* were observed. Plants were grown as described in Materials and Methods. Each replicate is a pool of one leaf from both the first and second leaf pair after infection (N=5 individual plants). Relative gene expression was measured with qPCR and calculated using the 2^-∆∆Ct^ method (Livak & Schmittgen, 2001) relative to the control condition (*GFP*-silenced plants) and normalized relative to the housekeeping gene, *SAND* (Pollier et al., 2014). ****p<0.0001, ***p<0.001, **p<0.01, *p<0.05 according to a t-test adjusted for FDR. Bar graphs represent the mean with error bars indicating the standard deviation


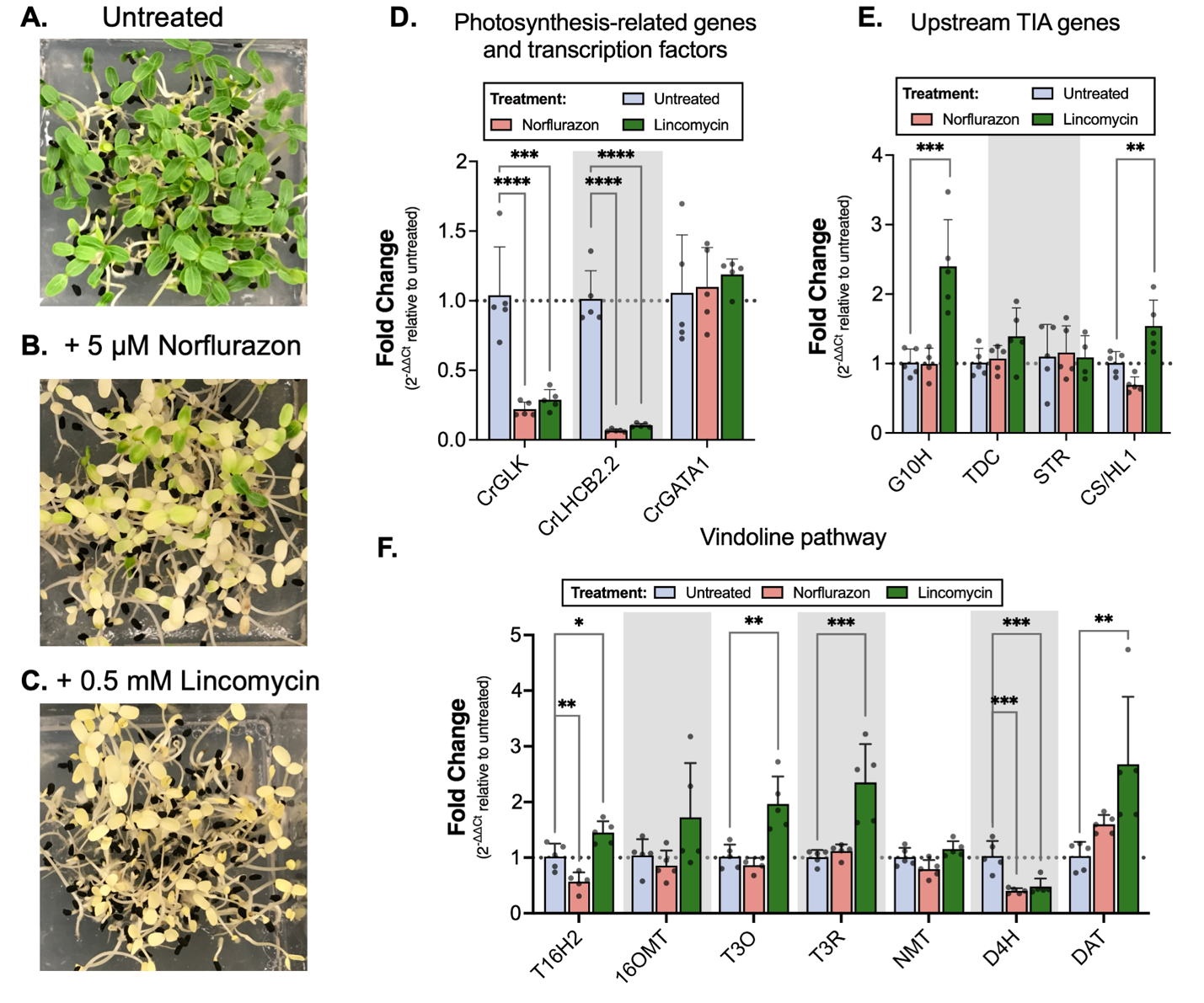


**Fig. S3** Lincomycin repressed the expression of *CrGLK* and *CrLHCB2.2* and activated the expression of most vindoline pathway genes in the presence of 1% sucrose. (A-C) Seeds were spread on Gamborg’s B5 media (+ 1% sucrose) containing 5 µM norflurazon (Nor) or 0.5 mM lincomycin (Lin). Nor was added from a stock solution dissolved in methanol (MeOH) while Lin was added from a stock solution dissolved in water. Compared to untreated seedlings, seedlings germinated in the presence of Nor or Lin showed inhibited chloroplast biogenesis after two full days of light exposure. (D) *CrGLK* and *CrLHCB2.2* expression were significantly inhibited by both Nor and Lin while *CrGATA1* expression was unaffected. (E) *G10H* and *CS/HL1* expression also increased with Lin treatment while *STR* and *TDC* expression was unaffected. (F) Many vindoline pathway genes increased in expression under Lin treatment, except for *D4H*, which decreased in expression under both Nor and Lin treatments. Relative gene expression was measured with qPCR and calculated using the 2^-∆∆Ct^ method (Livak & Schmittgen, 2001) relative to the mock condition, and normalized relative to the housekeeping gene, *SAND* (Pollier et al., 2014). Each replicate is a pool of 5 seedlings (N=5 replicates). ****p<0.0001, ***p<0.001 **p<0.01, *p<0.05 one-way ANOVA (adjusted for FDR) followed by a Dunnett’s test. Bar graphs represent the mean with error bars indicating the standard deviation


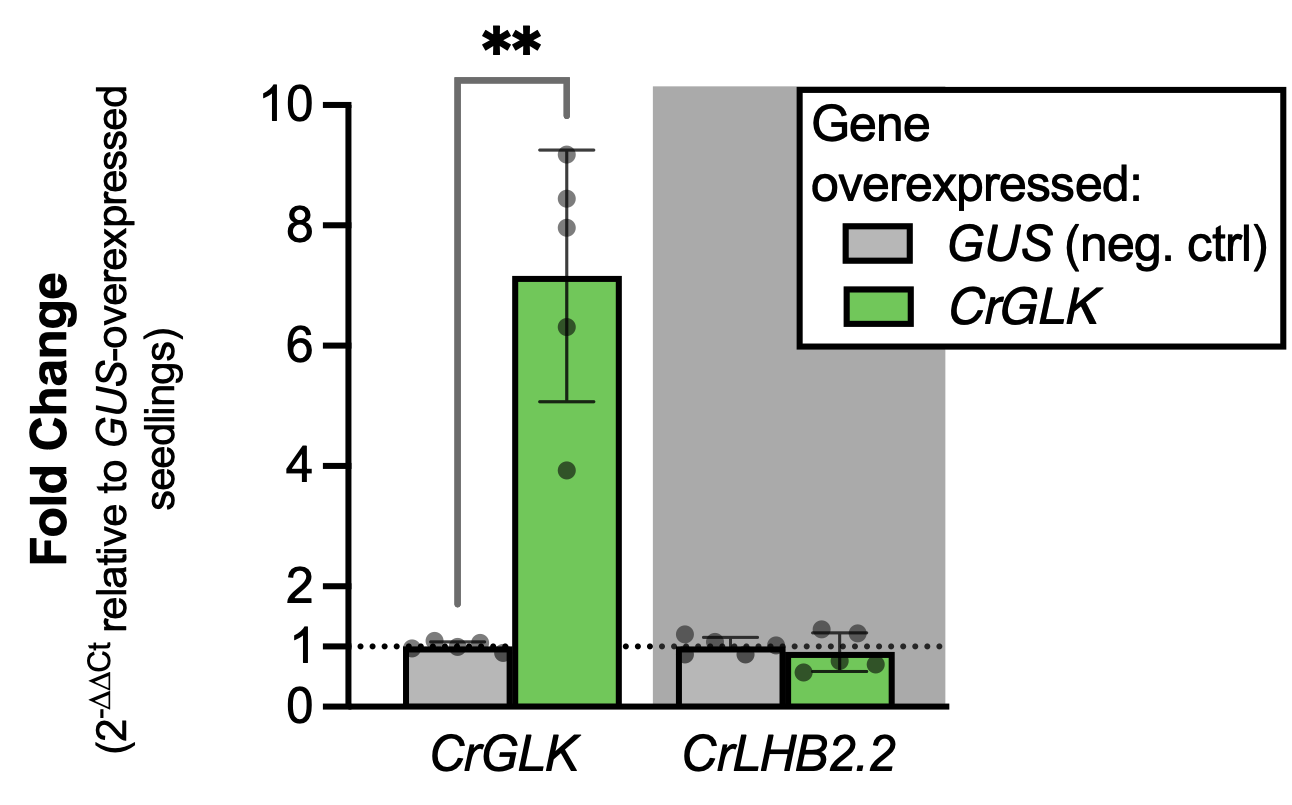


**Fig. S4** Overexpression of *CrGLK* in *C. roseus* seedlings was unable to increase *CrLHCB2.2* expression. *C. roseus* seedlings were infiltrated with *A. tumefaciens* carrying a *CaMV2x35S* driven *CrGLK* or a *CaMV2x35S* driven *GUS* (negative control). Relative gene expression was measured with qPCR and calculated using the 2^-∆∆Ct^ method (Livak & Schmittgen, 2001) relative to *GUS*-overexpressed and normalized relative to the housekeeping gene, *SAND* (Pollier et al., 2014). Each replicate is a pool of 5 seedlings (N=5 replicates). **p<0.01 according to a Welch’s t-test. Bar graphs represent the mean with error bars indicating the standard deviation

## Supplementary Methods

**Mass Spectrometry Analysis of Alkaloids**

Typical source conditions were set as follows: electrospray needle voltage 3500V; ion transfer tube temperature 300°C; vaporizer temperature 350°C. The nebulizer gas flow rates for sheath, auxiliary, and sweep gas were 50 arb, 10 arb, and 1 arb, respectively. The S-lens RF value used was 70 V, and the isolation window isolation window was set as 2 m/z. The automatic gain control (AGC) for the ions was set in standard mode, and the maximum injection time was set at auto for both full scan and MS/MS mode. The inclusion list contained the exact mass and the optimized collision energies. The optimal collision energies for each alkaloid standard compound were determined by direct infusion at a 10 uL/min flow rate. The collision energy was varied from 10 to 65 V to find the suitable collision energy. The full MS scan range was m/z 100–1000 with a resolution of 120,000. For MS/MS, the isolation window was set to 2 m/z with a resolution of 15,000.
